# Pre-exposure prophylaxis access, uptake and usage by young people: a systematic review of barriers and facilitators

**DOI:** 10.1177/20499361241303415

**Published:** 2024-12-07

**Authors:** Sarah Warzywoda, James A. Fowler, Amalie Dyda, Lisa Fitzgerald, Amy B. Mullens, Judith A. Dean

**Affiliations:** School of Public Health, Faculty of Medicine, The University of Queensland, 288 Herston Road, Brisbane, QLD 4006, Australia; School of Public Health, Faculty of Medicine, The University of Queensland, Brisbane, QLD, Australia; School of Public Health, Faculty of Medicine, The University of Queensland, Brisbane, QLD, Australia; School of Public Health, Faculty of Medicine, The University of Queensland, Brisbane, QLD, Australia; School of Psychology & Wellbeing, Centre for Health Research, Institute for Resilient Regions, University of Southern Queensland, Brisbane, QLD, Australia; School of Public Health, Faculty of Medicine, The University of Queensland, Brisbane, QLD, Australia

**Keywords:** HIV, HIV prevention, pre-exposure prophylaxis, young people

## Abstract

**Background:**

Young people’s sexual health decision-making, including decisions to access and adhere to HIV prevention strategies such as Pre-Exposure Prophylaxis (PrEP), are influenced by a range of internal and external factors. Synthesizing these factors is essential to guide the development of youth-focused PrEP health promotion strategies to contribute to international goals of ending HIV transmission.

**Objective:**

To understand the individual, interpersonal, sociocultural and systemic barriers and facilitators to PrEP access, uptake and use experienced by young people 24 years and younger.

**Design:**

A systematic review that adhered to the Preferred Reporting Items of Systematic Review and Meta-Analysis Protocols.

**Data Sources and Methods:**

Eight databases (PubMed, Scopus, Cochrane, Medline, CINAHL, JBI, EMBASE, Web of Science) were systematically searched using terms related to young people, HIV and PrEP use. A narrative synthesis approach was used to delineate key barriers and facilitators to PrEP access, uptake and use.

**Results:**

Of 11,273 returned articles, 32 met the eligibility criteria for inclusion: 18 from the United States, 10 from African nations and two from Brazil. Barriers and facilitators to PrEP access, uptake and use experienced by young people were identified across intrapersonal, interpersonal, community and systems levels. These factors are described under four overarching themes that relate to knowledge, side effects and perceptions of risk; attitudes and perceptions of family and partners; community attitudes and stigma; and negative healthcare provider experiences and difficulties navigating complex costly healthcare systems.

**Conclusion:**

Findings suggest individual-level factors need consideration alongside the impacts of healthcare systems and broader systemic sociocultural structures within young people’s relationships when developing PrEP health promotion strategies and services. Without considering these wider external implications to access, uptake and use of PrEP, global targets towards the elimination of HIV transmission will likely remain out of reach.

**Registration:**

This review was registered with Prospero (CRD42022296550).

## Plain language summary

Young people’s sexual behaviours and decision-making can be influenced by interpersonal factors including awareness, adherence, pill burden, and external factors such as parental attitudes/influence, peer/romantic/sexual relationships, community. This in turn can impact their HIV-related risks and prevention strategies they employ. This review aimed to understand the individual, sociocultural and systemic barriers and facilitators to HIV pre-exposure prophylaxis (PrEP) access, uptake and use experienced by young people ⩽24-years. Eight databases were systematically searched using terms related to young people, HIV, and PrEP use. Using a narrative synthesis approach data was delineated into key barriers and facilitators to PrEP access, uptake, and use. Of the 32 papers that met eligibility criteria for inclusion 18 were from the United States, 10 from African nations and two from Brazil. Barriers and facilitators to PrEP access, uptake and use experienced by young people were identified across the individual level, their relationships with family and partners, within the broader community and within healthcare systems. These factors are described under four overarching themes that relate to knowledge, side effects and perceptions of risk; attitudes and perceptions of family and partners; community attitudes and stigma; and negative healthcare provider experiences and difficulties navigating complex costly healthcare systems. Findings suggest individual-level factors need consideration alongside the impacts of health care systems and broader systemic sociocultural structures within young people’s relationships when developing PrEP health promotion strategies and services. Without considering these wider external implications to access, uptake and use of PrEP, global targets toward the elimination of HIV transmission will likely remain out of reach.

## Introduction

The transition from adolescence to young adulthood is one that encompasses an array of physical, psychological and social developmental changes.^1,2^ These changes include internal changes such as increased levels of self-sufficiency, autonomy and personal agency and external changes, such as evolving family/peer relationships, development of romantic or sexual relationships and greater exposure to community and cultural influences. This myriad of factors influences the development of a young person’s sexuality, sexual health decision making and sexual behaviours (including HIV-related risk behaviours).^1,3,4^ They can also contribute to increased HIV risk by reducing a young person’s ability to seek HIV information and access prevention strategies, including HIV pre-exposure prophylaxis (PrEP).^4^ This is of concern when evidence indicates younger people already have lower awareness and knowledge of, and uptake and adherence to, HIV prevention strategies.^5–8^

Despite global improvements in the scale-up of HIV testing, treatment and prevention, young people remain disproportionately affected by the HIV epidemic worldwide. Young people aged 15–24 years make up 22% of the global population and accounted for over 36% of all new HIV notifications in 2022.^9–12^ Despite this disproportionate burden, PrEP uptake within this group is lagging in many regions.^13–18^ Understanding factors that impact PrEP access, uptake and use in young people requires a holistic approach encompassing the nuanced understanding of individual, sociocultural and structural barriers that young people experience. This understanding can be garnered through the use of various frameworks such as the socioecological model (SEM) – which can be useful to explore the array of factors that can influence PrEP access and uptake and the interactions across the various levels of an individual’s life experiences.^19^ In previous applications of the SEM within the HIV context these levels have been identified as synergistically influencing PrEP access in adults from priority populations.^19^ Understanding these factors across an individual’s intrapersonal (individual), interpersonal (parents, partners, peers), community and structural (healthcare systems) experiences – particularly among priority subgroups (e.g. young MSM, young women) – is imperative in guiding successful interventions that improve access and uptake of HIV prevention methods.^9,20^

Provision of PrEP to young people has expanded in many regions (e.g. United States, Australia, Canada, France, Kenya, Eswatini),^21–26^ but to ensure young people have equitable access to PrEP, a nuanced understanding of their barriers and facilitators to PrEP access, uptake and use is essential. The purpose of this mixed-method systematic review was to conduct a narrative synthesis of the individual, interpersonal, systemic, and sociocultural barriers and facilitators to PrEP access, uptake and use experienced by young people ⩽24 years.^27^ To our knowledge, this is the first article to review PrEP access and uptake experiences with young people globally. These findings will delineate strategies to ensure young people are not left behind in the race towards global fast-track targets to end the HIV epidemic by 2030.^28^

## Methods

### Review registration

The review was registered with Prospero (PROSPERO 2022 CRD42022296550) to investigate two research questions: (1) What are the patterns of PrEP use among young people aged ⩽24 years? and (2) What are the factors influencing PrEP access, uptake and patterns of use among young people aged ⩽24 years? Upon reviewing the search yields, research question 1 was not explored given inadequate reporting of patterns of use among young people aged ⩽24 years globally.

### Search strategy

This review adhered to the Preferred Reporting Items of Systematic Review and Meta-Analysis Protocols (PRISMA). The initial search strategy was devised by three authors (SW, JD, JF) in consultation with university library staff experienced in systematic searching to refine search terms relating to young people, HIV and the use of PrEP (Supplemental Table 1). Eight databases were systematically searched (PubMed, Scopus, Cochrane, Medline, CINAHL, JBI, EMBASE, Web of Science) in January 2022 and were re-run in May 2024. To further identify potential studies, backwards and forwards reference search of the included studies was conducted.

### Inclusion criteria and study selection

To align with international definitions of young people used by The Joint United Nations Programme on HIV/AIDS (UNAIDS) and the World Health Organization (WHO),^29,30^ articles that discuss PrEP access, uptake and use in young people aged 24 years and younger were included. To be eligible articles needed to specifically stratify results to young people 24 years and younger. Studies published in peer-reviewed journals, written in English, and encompassing all study methodologies were included.

Endnote 20^31^ was used to combine database searches. Title and abstract reviews were completed by the first author (SW), with a random review of 10% by a second author (JF) to ensure reliability and consistency of paper inclusion. Discrepancies were resolved through group deliberation. Full-text review was completed by the first author (SW), with all excluded articles checked by the second reviewer (JF).

### Data extraction, analysis and quality assessment

A narrative synthesis approach was used to systematically review and synthesise the qualitative and quantitative data using a textual approach in order to ‘tell the story’ from the findings of the included articles.^32^ The analysis was guided by the methods described by Popay et al.^32^ A data extraction table was used to extract descriptive details from each study and identify the key barriers and facilitators from each paper to help determine similarities across the studies. A preliminary analysis was then used to code the similarities across four components of the SEM – intrapersonal/individual (knowledge, attitudes and perceptions), interpersonal (parents and partners), community (stigma and social support) and structural (healthcare systems)^19^ – and was reviewed by all authors with all discrepancies deliberated as a team. A narrative synthesis using a thematic analysis approach was then used to develop groupings of similar codes to form an understanding of how each component influenced PrEP access, uptake and use independently and simultaneously within young people.

The included studies were critically appraised using the Mixed Methods Appraisal Tool (MMAT) version 2018.^33^ The MMAT tool provides a validated quality appraisal tool for reviews that include multiple study designs including qualitative, quantitative and mixed-methods.^33^ The overall quality rates for each study are provided in Supplemental Table 2.

## Results

**Figure 1.**
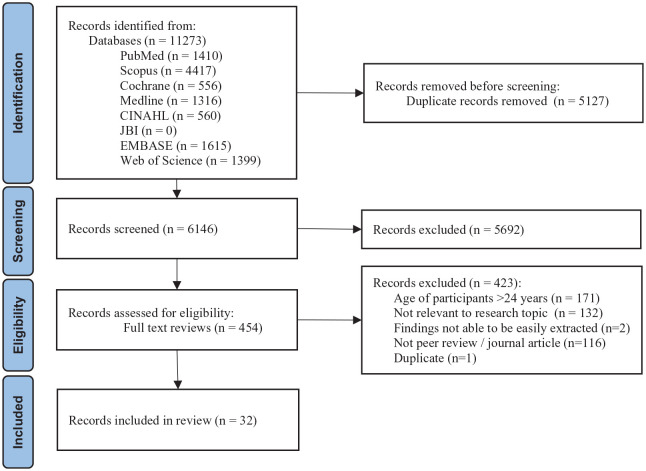
PRISMA flow diagram. PRISMA, preferred reporting items for systematic reviews and meta-analyses.

Initial searches yielded 8837 articles, and the updated search identified an additional 2936 (total yield = 11,273), which were refined to 32 articles from 23 separate studies/programmes (Figure 1). Table 1 summarises the descriptive details of the included articles. Eighteen of the articles were from the United States and 12 in countries across the Africa region (including Kenya (8), South Africa (3), Uganda (2), Zimbabwe (1), Namibia (1) and Tanzania (1)) and two from Brazil. Eighteen articles reported findings that used qualitative methods (e.g. in-depth interviews, focus groups),^34–51^ eight used quantitative methods (e.g. cross-sectional surveys, randomised controlled trial)^52–59^ and six articles were multi-methods/mixed-methods designs.^60–65^

**Table 1.** Characteristics of included articles.

Author (year)	Country	Study design	Study/programme	Aim^a^	Participant details	Study findings
Qualitative articles						
Atujuna et al. (2021)	South Africa	Qualitative interviews	iPrevent	Explore family influence AYAs’ approach towards and use of PrEP	– 18–24 years – Male and female^b^ – Self-identify as heterosexual and MSM	– PrEP use was influenced by family support; family attitudes; family disclosure, and other family members using PrEP – Dimensions of family closeness (i.e. close, in-between and loose-knit) were important in contextualizing family influence on PrEP use
Baron et al. (2020)	South Africa and Tanzania	Qualitative interviews	EMPOWER	Understand the role and benefits of peer-based clubs incorporating an empowerment curriculum for AGYW taking PrEP	– 16–24 years – Female – Sexual orientation not stated	– Club participants reported increased self-esteem and self-efficacy, reduced isolation, greater insight and strategies to address gender-based violence – Clubs provided a safe space for sharing problems and provided strategies to improve partner communication
Birnholtz et al. (2021)	US	Qualitative interviews		Exploring gay and bisexual MSM’s knowledge and perceptions of PrEP, and the barriers they perceive	– 15–19 years – Male – Self-identified as MSM	– Despite PrEP awareness participants were unsure of insurance coverage and out-of-pocket costs – Participants felt parents and providers would not be knowledgeable or supportive – Participants were reluctant to share their use of PrEP on social media
Camlin et al. (2020)	Kenya and Uganda	Qualitative interviews	SEARCH	Deepen the understanding of PrEP demand and early uptake among young women and men	– 15–24 years – Male and female – Sexual orientation not stated	– HIV severity was perceived as low, uptake was motivated by high perceived HIV risk, and beliefs that PrEP use supported life goals – Men viewed PrEP as helping to safely pursue multiple partners – Women felt they had to ask male partners permission to use PrEP, and saw PrEP as a way to control risks relating to transactional sex and limited agency to negotiate condom use
Crooks et al. (2023)	US	Open-ended cross-sectional survey		Explore barriers to PrEP uptake experienced by Black girls and women in Chicago, US	– 13–24 years – Female – Sexual orientation not stated	– Content analysis identified barriers to PrEP uptake including side effects, financial concerns, medical mistrust, lack of PrEP knowledge and misconceptions, stigma, privacy concerns
Gailloud et al. (2021)	US	Qualitative interviews		Inform the development of adolescent-specific strategies to make PrEP more accessible	– 15–17 years – Male and female – Self-identified as heterosexual or bisexual	– PrEP awareness was low; however, the majority were enthusiastic when informed and felt it empowered them to have control over their health. – Multiple barriers were identified, including confidentiality from parents low perceived need, concerns about adherence and side effects – School-based health centres were considered trusted sources of confidential, accessible care
Hartmann et al. (2021)	Kenya	Intervention design	Tu’Washindi na PrEP	Describe the participatory process used to develop and refine the locally relevant multilevel intervention,	– 15–24 years – Female – Sexual orientation not stated	– Barriers to PrEP use that were considered for intervention development included; individual (e.g. knowledge, confidence, personal agency); interpersonal (gender roles and intermate partner violence); Service provision (provider judgement/stigma); Community (Poverty, unintended pregnancy, inequitable gender norms, stigma)
Hess et al. (2019)	US	Qualitative interviews	‘‘Good to Go’’ Programme for HIV testing	Investigate reasons for not using PrEP among YMSM accessing to HIV testing services	– 18–24 years – Male – Self-identify as MSM	– Barriers to PrEP included daily bill burden, low perceived risk, side effects, stigma, social or provider influence on decisions, preference for current prevention strategy
Marsh and Rothenberger (2019)	US	Case report		Case report of a young black MSM who accessed PrEP however acquired HIV due to cessation of use	– 18 years – Male – Self-identified as MSM	– The young man was able to successfully access PrEP but was unable to adhere to the regimen and engage in follow-up care, ultimately acquiring HIV – Barriers to adherence were difficulty swallowing large tablets
McKetchnie et al. (2023)	US	Qualitative Interviews		Explore barriers and facilitators to PrEP among YMSM and their perspectives on peer navigation to improve uptake/adherence	– 17–24 years – Male – Self-identified as MSM	– Multiple factors influence PrEP uptake/adherence including perceived costs, anticipated stigma, sexual activity, relationship status – establishing pill-taking routines is an important adherence facilitator; and peer navigators could offer benefits for PrEP adherence
Muhumuza et al. (2021)	Uganda, Zimbabwe and South Africa	Qualitative interviews and FGD	CHAPS	Explore barriers and facilitators to uptake of PrEP among adolescents/young people, to inform PrEP implementation	– 13–17 and 18–24 years – Male and female – Sexual orientation not stated	– Barriers included individual factors (fear, side effects); interpersonal (parental influence, sexual relationships); community (peer influence, stigma); institutional (clinic wait times, provider attitudes); structural (cost, modality, accessibility) – Facilitators included individual factors (high risk perception); interpersonal (peer influence, social support); community (adequate PrEP information, efficacy/safety); institutional (convenient/responsive and appropriate services); structural (access/availability, costs)
Pintye et al. (2021)	Kenya	Qualitative interviews	PrIYA Programme	Evaluate modifiable factors that impede PrEP use among women receiving PrEP within maternal and child health and family planning clinics	– 15–24 years – Female – Sexual orientation not stated	– PrEP use/adherence was facilitated by encouragement from close confidants – Pregnancy helped conceal PrEP use due to normalised pill-taking during pregnancy, concealment became more difficult postpartum – Frequently testing HIV-negative reassured AGYW of PrEP efficacy and motivated persistence
Rogers et al. (2021)	Kenya	Qualitative interviews	PrIYA Programme	Understand factors influencing PrEP decision-making among AGYW to inform tailored PrEP implementation strategies	– 15–24 years – Female – Sexual orientation not stated	– Known or suspected partner infidelity motivated use however potential partner reactions was a barrier – Among pregnant AGYW, the responsibility of motherhood staying healthy and remaining HIV-free, was a strong motivator – Fears of negative impacts on fertility or reductions in contraceptive effectiveness led to declining PrEP. – Supportive peers facilitated by PrEP decision-making
Santos et al. (2023)	Brazil	Qualitative interviews	PrEP1519 study	Explore the PrEP perceptions and experiences of young GBMSM, considering the intersecting social markers of difference and how they constitute barriers and facilitators	– 16–20 years – Assigned male at birth – Self-identified as MSM	– Willingness to use and adhere to PrEP is part of a learning process, production of meaning, and negotiation in relation to HIV/STIs and the possibilities of pleasure – Accessing and using PrEP makes several adolescents more informed about their vulnerabilities, leading to more informed decision-making
Shorrock et al. (2022)	US	Qualitative interviews	PUSH Study	Using an ecological framework examine the lived experiences of PrEP barriers among young Black and Latinx SMM and TW	– 17–24 years – assigned male at birth – MSM and TGW	– Barriers were identified across the individual, family, community and structural level including low perceived HIV risk, fear of disclosure, stigma, barriers relating to insurance/cost and medication use – Partners with HIV encouraged PrEP use
Vera et al. (2023)	Kenya	Qualitative interviews		Understand AGYW experiences with pharmacy-based PrEP, reasons for preferring pharmacy-based PrEP delivery	– 15–24 years – Female – Sexual orientation not stated	– AGYW preferred pharmacies for accessing PrEP and were willing to pay for PrEP even if available for free at healthcare clinics – Reasons for pharmacy preference included accessibility, lack of queues, and medication stockouts, privacy, anonymity, autonomy, and high-quality counselling from study nurses
Zapata et al. (2021)	US	Cross-sectional online FGD		Explore the perceived impact of the COVID-19 pandemic on HIV prevention among young sexual minority men	– 17–24 years – Male (including transgender men) – Self-identify as MSM	– Negative effects of COVID-19 pandemic causing limited and disrupted access to HIV testing, PrEP, and post-exposure prophylaxis, and lack of appropriate services – PrEP barriers were compounded by COVID-19-related challenges including relocating back home with family needing to concealing identity/PrEP use; fears COVID-19 by attending clinical appointments
Quantitative articles						
Bonett et al. (2021)	US	Randomised controlled trial	PUSH	Explore how economic vulnerability, sexual network-related factors, and individual HIV risk are associated with the PrEP continuum	– 15–24 years old – Male – Self-identified as MSM	– High willingness/intention to use, yet 82% not currently taking PrEP. – Health insurance (aOR = 2.95, 95% CI = 1.60–5.49), having ⩾1 PrEP users in sexual network (aOR = 4.19, 95% CI = 2.61–6.79), and higher HIV risk scores (aOR = 1.62, 95% CI = 1.34–1.97) were associated with being further along the PrEP continuum
Hong et al. (2021)	US	Cross-sectional survey		Examine how COVID-19 and associated public health measures affected sexual behaviour and PrEP use among YSMM	– 17–24 years – Male (including transgender men) – Self-identified as MSM	– 15% of PrEP users discontinued use during COVID-19 and reported decreased sexual activity – 20% reported difficulty getting prescriptions/medications from doctors or pharmacies – Among those who met CDC PrEP criteria 86.5% were not using PrEP
Macapagal et al. (2020)	US	Cross sectional survey		Describe PrEP awareness, use, and perceived barriers among adolescent MSM	– 15–17 years – Male – Self-identified as MSM or same sex attracted	– Awareness of PrEP (54.8% of participants) was associated with older age, having used GSN applications, and greater HIV knowledge – Being unsure how to access PrEP (56.1% of participants) was associated with more partners, lower HIV knowledge, and never having talked to a provider about PrEP – Believing that one could not afford PrEP was predicted by greater perceived risk of HIV
Moskowitz et al. (2021)	US (incl. Puerto Rico)	Cross-sectional survey	SMART	Our study aims to explore where adolescent MSM fall on the Motivational PrEP Cascade	– 13–18 years – Assigned male at birth. – Self-identified as MSM	– 53.9% were identified as eligible PrEP candidates. Of those identified as appropriate only 16.3% of candidates reached preparation (stage 3; seeing PrEP as accessible and planning to initiate PrEP) and 3.1% reached PrEP action (stage 4; prescribed PrEP) – Factors associated with reaching later stages were being older, being out to parents, and engaging in previous HIV/STI testing
Sila et al. (2020)	Kenya	Cross-sectional survey (quant)	PrIYA Programme	Evaluate psychosocial characteristics, behavioural risk factors for HIV, and PrEP awareness and uptake among AGYW seeking family planning services	– 15–24 years – Female – Sexual orientation not stated	– 89% of AGYW were aware of PrEP; 76% had at least one PrEP eligibility criterion as per national guidelines; only 4% initiated PrEP – PrEP initiators more frequently had high HIV risk perception than non-initiators (85% vs 10%, *p* <0.001) – Low perceived HIV risk (76%) and pill burden (51%) were common reasons for declining PrEP
Tapsoba et al. (2021)	Kenya	Cohort study	DREAMS	PrEP persistence among AGYW who initiated PrEP as part of the DREAMS programme	– 15–24 years – Female – Sexual orientation not stated	– PrEP programme persistence varied by county (*p* < 0.001), age at PrEP initiation (*p* = 0.002), marital status (*p* = 0.008), transactional sex (*p* = 0.002), GBV experience (*p* = 0.009) and current school attendance (*p* = 0.001)
Tapsoba et al. (2022)	Kenya	Prospective study	DREAMS	Extent of programme persistence and the level of protection from HIV infection among programme attendees	– 18–24 years – Female – Sexual orientation not stated	– Among AGYW perception of being at moderate-to-high risk for HIV if not taking PrEP was associated with persistence (aOR, 10.17 [95% CI 5.14 to 20.13], *p* < 0.001) – >90% who continued PrEP indicated they were using PrEP to prevent HIV, although almost all had non-protective TFV-DP levels
Whitfield et al. (2020)	US	Cross-sectional survey		Understand the prevalence of and factors associated with PrEP use among a large sample of young and adult sexual minority men	– 13–24 years – Male – Self-identify as MSM	– Older age was positively associated with both former and current PrEP use – YSMM who identified as gay (vs bisexual), lived in the Northeast, Midwest, and West (vs South), had their own health insurance (vs those on their parent’s), had recently been diagnosed with an STI, and had recently used a drug all had higher odds of being a current PrEP user
Zeballos et al. (2022)	Brazil	Cohort study	PrEP1519 study	Describe PrEP discontinuation among MSM and TGW and to investigate the associated factors for PrEP discontinuation	– 15–19 years – Self identify as MSM or TGW	– Multivariate analysis demonstrated that TGW (aHR = 1.63; 95% CI: 1.02–1.64) and adolescents with a medium (aHR 1.29; 95% CI: 1.02–1.64) or low (aHR 1.65; 95% CI: 1.29–2.12) perceived risk of HIV infection had an increased risk of discontinuation, whereas the adolescents with a partner living with HIV had a lower risk of discontinuation (aHR 0.57; 95% CI: 0.35–0.91)
Multi-methods articles						
Barnabee et al. (2022)	Namibia	Mixed-methods	DREAMS	Explore whether and how PrEP service delivery through community and hybrid community-clinic models results in improved PrEP persistence among AGYW	– 15–24 years – Female – Sexual orientation not stated	– In the community and hybrid models, PrEP persistence was related to: – Individualised service delivery offered refill convenience/simplicity – Consistent interactions and shared experiences fostered social connectedness with providers and peers. PrEP/HIV-related stigma was widely experienced outside of these networks – Referral to unfamiliar PrEP services and providers for PrEP refill triggered apprehension and discouraging use
Horvath et al. (2019)	US	Multi-methods	Project Moxie	Describe pre-exposure prophylaxis (PrEP) awareness, willingness to use PrEP, barriers to facilitators of PrEP uptake, and PrEP	– 15–24 years – transgender and gender nonbinary (TGNB) – 75% self-identified as LGBQ	– Despite PrEP awareness most perceived low HIV risk resulting in low PrEP interest – Barriers to PrEP utilisation included cost, previous negative experiences of medical institutions, medical mistrust, concerns about disclosure, concerns about hormone interaction and insurance coverage
Moskowitz et al. (2020)	US	Mixed-methods	SMART	Better understand the role of parents in adolescents’ attitudes towards PrEP	– 13–18 years – Assigned male at birth – Self-identified as MSM	– Most perceived parents would be unsupportive of PrEP and would likely be angry, accusatory, and punitive if PrEP use was discovered – Accessing PrEP independent of parents was thought to increase health autonomy, agency, and prevent awkward conversations about sex. – Low self-efficacy to communicate with parents about PrEP contributed to participants feeling PrEP was not ‘right’ for them, resulting in lower PrEP interest
Owens et al. (2021)	US (incl. Puerto Rico)	Mixed-methods	SMART	Understand factors that either facilitate or hinder engaging in PrEP follow-ups and understand ASMM’s beliefs about PrEP follow-up appointments	– 13–18 years – Assigned male at birth – Self-identified as MSM	– 73.0% had heard about PrEP, 45.3% were unsure if PrEP was right for them and 50.4% were unsure if they intended to take PrEP. – Older age was associated with greater confidence in being able to attend follow-up appointments – Barriers included fear of ‘outing’ oneself to parents, concealing appointments, costs, insurance, reliance on parental transport
Wood et al. (2019)	US	Mixed-methods		To discover barriers and facilitators of PrEP adherence in young transgender women and MSM of colour	– 15–24 years – Assigned male at birth – MSM and TGW	– Adherence barriers included stigma, health systems inaccessibility, side effects, competing stressors, and low HIV risk perception. – Facilitators included social support, health system accessibility, reminders/routines, high HIV risk perception, and personal agency.
Wood et al. (2020)	US	Mixed-methods	PrEP Together	To characterise perceived social support for young men and transgender women who have sex with men taking PrEP	– 15–24 years – Assigned male at birth – MSM and TGW	– Participants characterised support as instrumental (e.g. transportation); emotional (e.g. affection); and social interaction (e.g. taking medication together) – Key characteristics of PrEP support figures included closeness, dependability, and homophily (alikeness) with respect to sexual orientation

AGYW, adolescent girls and young women; aOR, Adjusted Odds Ratio; aHR, Adjusted Hazard Ratio; ATN, Adolescent Medicine Trials Network; CHAPS, Combined HIV Adolescent PrEP and Prevention; CI, Confidence Interval; DREAMS, Determined, Resilient, Empowered, AIDS-free, Mentored and Safe; EMPOWER, Enhancing Methods of Prevention and Options for Women Exposed to Risk; FGD, focus group discussion; GBMSM, gay bisexual and other men who have sex with men; GBV, gender-based violence; MSM, men who have sex with men; PrEP, Pre-Eposure Prophylaxis; PrIYA, PrEP Implementation for Young Women and Adolescents Programme; PUSH, Providing Unique Support for Health; RCT, Randomised control trial; SEARCH, The Sustainable East Africa Research in Community Health; SMART, Sequential Multiple Assignment Randomised Trial; TFV-DP, tenofovir diphosphate; TGW, Transgender women; US, United States of America.

^a^Aims have been condensed from original study aims for the purposes of this table.

^b^We used study definitions of gender and may or may not be inclusive of trans (binary and non-binary) identities.

Overall, five of the quantitative articles reported that young age was significantly associated with lower PrEP awareness, use and persistence. This was observed across all age groups when comparing young people to older adults (younger than 18/19 years compared to those ages 19/20 and older)^52,57^ and within groups of young people (14/15 year olds compared to 18 year olds).^54,55,58^ Thematic analysis of the 32 studies provides nuance to this finding that young people are significantly less likely to be aware of, and adhere to, PrEP for HIV prevention, and delineates the socioecological challenges and supports impacting young people. These themes and their identified subthemes are described in detail below and presented in Table 2. Quotes selected from the qualitative articles are used to provide context and voice to young people.

**Table 2.** Summary of narrative synthesis on the barriers and facilitators to access, uptake and use of PrEP in young people.

Theme/sub-theme	Barriers	Facilitators
Knowledge, perceptions and experiences influence PrEP use		
*Is PrEP for us and is it worth the hassle? – knowledge perceptions, and experiences of young people influence PrEP use*		
	• Lack of awareness of PrEP and uncertainty of efficacy • Concerns of reduced efficacy of hormonal contraceptives and affecting pregnancy outcomes and breastfeeding • Lack of representation of young people in advertisements and endorsements • Concerns and experiences of side effects • Aversion to and difficulties taking pills • Inconvenience of follow up appointments, tests and daily pill taking	• User experience and testing negative at follow up appointments improved normalisation of use, awareness of use • Incorporating PrEP into current routine, for example,with other medications helped adherence • A treatment buddy, someone to take pills with or partner who is on antiretroviral therapy (ARV) for HIV treatment helped with adherence
*Not ‘risky’ enough to take PrEP – perceptions of HIV risk impacts PrEP uptake*		
	• Not considering themselves to be at enough risk • PrEP associated with being ‘promiscuous’, having multiple sexual partners or engaging in ‘risky’ sexual practices • Not feeling scared of HIV and expressed greater fears of other issues such as accidental pregnancy, or cancers	• Higher perception of risk, combined with higher levels of HIV knowledge and engagement with STI/HIV services • Partners having sex outside the primary relationship increased the perceived risk of HIV, prompting PrEP use for their personal protection
Gatekeepers versus cheerleaders – the impact of interpersonal relationships on PrEP use		
*The ‘freedom’ to use PrEP – Family attitudes led to concealment or support for PrEP use*		
	• Parental and family concerns about PrEP efficacy and side effects • Parental concerns and perceptions of young people having sex under the age of 18 or before marriage • Misperceptions that PrEP was used to ‘*sleep around*’, is an illegal substance, or used for HIV treatment • Fears of parental repercussions/punishment for sexual identity and behaviours, having to ‘out’ themselves to parents to access PrEP • Prevented from using PrEP (e.g. discouraging use, confiscating, throwing pills out)	• Being able to discretely take PrEP, for example, transferring the pills into another bottle • Family members supporting appointment attendance, list a family member as clinic contact, and have family provide pill reminders. • Support from parents of sexual orientation/behaviours enabled comfort in discussing PrEP and encouraged other family members to seek PrEP • Parental support helped young women in African regions conceal PrEP from unsupportive partners
*Do I need my partners permission to use PrEP?*		
	• Accusations of infidelity, scepticism/misperceptions about partner’s HIV status • Traditional power imbalances or normative gender roles and partners engaging in practices such as concurrent sexual partners, polygamy and transactional sex • Partners controlling condom use, experiences of physical violence and reports of partners hiding, confiscating or discarding PrEP pills	• Future plans (finishing school or having a family) • Remain healthy and alive to look after their current children or prevent vertical transmission during pregnancy • Taking PrEP with a partner to protect each other • Privacy/discretion when taking PrEP, for example, transferring PrEP medication into another bottle or convincing partners its medication for pregnancy
Communities can discourage use – community stigma can be overcome by supportive peers		
	• Community attitudes and peer disapproval/judgement • Fearful of people thinking ‘*you have the disease [HIV]*’ • Fears of judgement and rumours regarding pill recognition, being seen attending clinics, or judgement of sexual behaviour that PrEP is being taken to ‘*sleep around*’, being labelled a ‘*whore*’ or ‘*dirty’*	• Social clubs/groups and peer support provided connection and shared experiences, motivation and support for PrEP uptake and continuation • Social/peer support helped participants hide PrEP use from unsupportive partners • A treatment buddy provided encouragement and adherence reminders • Community use and positive attitudes aided in normalising, encouraging and empowering young people to use PrEP and attending clinics • Rejection of stigma enabling personal agency and autonomy
The healthcare system paradox – the healthcare system itself limits healthcare access		
	• Access difficulties including lack of proximity to healthcare providers/pharmacies offering PrEP and a need to rely on parents transportation to access these clinics, clinic closures, long clinic wait times, health centres running out of medications • Lack of gender and sexuality affirming healthcare providers • 3-monthly follow-up appointments were inconvenient, unmanageable, and resulted in scheduling conflicts (e.g. school, extracurricular activities, unanticipated events) • Confidentiality concerns about providers disclosing PrEP use to parents • Cost of PrEP restricts consistent access (e.g. medications, travel, healthcare appointments, unexpected financial strain due to unstable employment/ living costs, and inability to afford PrEP without parental health insurance)	• Proximity to affordable local clinics, school-based clinics and affirming care • School/college-based health clinics that assured confidentiality, and no parental involvement • Providing PrEP at no cost and at local clinics/pharmacies
*HCP practices and attitudes influence care – Negative experiences led to medical mistrust*		
	• Negative experiences with healthcare providers, including judgement or discrimination of participants ‘lifestyle’, sexual orientation, or number of sexual partners • Lack of HCP knowledge, lack of supportive and affirming care • HCP not providing PrEP to bisexual men	• PrEP adherence counsellors and regular reminders helped young people maintain adherence. • Trust in HPC • Being offered practical advice on side effect management, discontinuation, missed dosages and re-initiation

HCP, healthcare providers; PrEP, pre-exposure prophylaxis.

### Knowledge, perceptions and experiences influence PrEP use

Fifteen qualitative articles^35–47,61,65^ and five of the quantitative articles,^53,54,56,59,63^ discussed the intrapersonal factors that impact PrEP access, uptake and use in young people. These factors included knowledge and awareness of PrEP, inconvenience, pill burdens, and perceptions of risk.

#### Is PrEP for us and is it worth the hassle? – Knowledge perceptions, and experiences of young people influence PrEP use

A lack of awareness of PrEP and uncertainty around its efficacy was reported in four articles involving young people from the United States.^38,39,41,61^ and six articles from African nations.^35,37,40,44–46^ Two quantitative articles involving young people from the US found reluctance to take PrEP was associated with lower HIV knowledge,^54^ and lower PrEP awareness.^54,63^ Young people reported uncertainty of PrEP efficacy and perceived condoms to be more effective.^39,41,46^ Young women from African nations raised concerns about PrEP reducing the efficacy of hormonal contraceptives, and misconceptions about the effects of the ‘*chemicals*’ in PrEP on pregnancy outcomes and breastfeeding.^35,45,46^ Young women in the US reported fears that PrEP may interfere with natural adolescent development or worsen pre-existing conditions.^38^ A lack of representation of young people, and specific populations of young people in advertisements and endorsements, reinforced the idea that PrEP is not for them.^36,44^ For example, young women in the US described not being aware that PrEP was available to women.^38^ Young people reported that PrEP use by peers seen through social media was a motivator for use.^43^

. . . .I think it requires campaigns to be done in the population like you moving around encouraging young people to take PrEP and you people telling us why it’s good. (female 21 years; Zimbabwe)^44^

Confidence in PrEP efficacy and normalisation of its use was gained by young people across Africa, through user experience, testing negative at follow-up appointments^37,45,46^ and awareness of use and testimonies from within the community.^37,46^ However, young people from across these settings described aversion to taking pills^39,42,44,45^ and difficulties swallowing large PrEP pills.^39,42,44,45^ Sila et al. (2020)^56^ reported among their cohort of adolescent girls and young women in Keyna, that 51% declined PrEP due to pill burden. The need for periodic follow-up appointments resulted in PrEP being considered a ‘*burden’* or a ‘*hassle’* not worth the effort.^36,39,41,44,45^ For others, there were anticipated struggles with adherence due to forgetfulness^39,45^; lack of a daily routine or inconsistent schedules^39,49^; being away from home^39,44,49^; limited privacy to take pills^44^; and changes in circumstances.^39,45^ Incorporating PrEP into one’s routine (e.g. with other medications) or using phone reminders (although participants reported these could be easily ignored) were reported to facilitate adherence.^43,45,47^ Macapagal et al. (2020)^54^ reported that a reluctance to use PrEP due to fear of side effects was significantly associated with lower HIV knowledge, and never having heard of PrEP before their study. Additionally, experiences of side effects (including dizziness, fatigue, nausea, diarrhoea) led to discontinuation.^37,39–41,44–46,47,49, ^54^,61,65^

Even if you just bring tablets and put them there, I just vomit. Some may fear to take PrEP tablets and say that “I rather fall sick with the thing [HIV] than taking those tablets.” (female 13–17 years; Uganda)^44^

#### Not ‘risky’ enough to take PrEP – Perceptions of HIV risk impacts PrEP uptake

Perceptions surrounding personal risk were discussed in fifteen of the articles across the three contexts (African,^37,44,45,49,56^ the United States^36,41,43,53–55,61,65^ and Brazil^47,59^). Barriers to use and discontinuation were significantly associated with low perceived HIV risk^54,56,59^ and reductions sexual behaviours – despite Hong et al.^53^ reporting that 53.5% still meet national guideline criteria for PrEP. In qualitative findings PrEP use was associated with perceptions of being ‘*promiscuous*’ and only needed for those with multiple sex partners or engaging in risky sexual practices.^36,41,44,45,47,6^ Young people from Kenya and Uganda described not feeling scared of HIV and expressed greater fears of other things such as accidental pregnancy or cancer.^37^

Some youth now days do not see HIV/AIDS as a serious disease, just because they know there is ARVs [antiretrovirals]. Some youths say, “even if I contract HIV I will go to [the] health center and start taking ARVs.”. . .For girls, they are mostly scared about pregnancy and the boys are only scared of being imprisoned for having impregnated a girl. (male; 15-24 years; Uganda)^37^

A higher perception of risk, having higher numbers of sexual partners and engagement with STI/HIV testing lead to increased PrEP awareness and uptake.^43,47,49,54–56,58,65^ For young women in Kenya, Uganda, Zimbabwe and South Africa, partners having sex outside the primary relationship increased the perceived risk of HIV, motivating them to seek PrEP for their personal protection.^35,37,44,49^

### Gatekeepers versus cheerleaders – The impact of interpersonal relationships on PrEP use

Fifteen qualitative^34–37,39–41,44–48,51,62,63^ and eight quantitative articles^52,54,55,56,57,59,62,63^ provided findings on the positive and negative impacts of interpersonal relationships (family/partners) on attitudes and perceptions towards PrEP that act as ‘cheerleaders’ and facilitators by promoting a sense of acceptance and support or ‘gatekeepers’ and barriers that fostered attitudes, perceptions and environments where young people felt pressure to conceal use due to fear of judgement and harm.

#### The ‘freedom’ to use PrEP – Family attitudes led to concealment or support for PrEP use

Young people from both the United States^36,39,51,62,63,65^ and African settings^34,35,37,44–46^ reported parental and family concerns of PrEP use, including efficacy and side effects,^34^ having sex under 18-years or before marriage,^44,^62^^ misperceptions that PrEP was used to ‘*sleep around*’,^37,62,63^ that PrEP was an illegal substance or believed that it was used for HIV treatment (not prevention).^34,35,62^ Fears about repercussions and punishment for sexual identity and behaviours,^62,63^ or physically being prevented from using PrEP (e.g. confiscating pills, throwing pills out)^34,37,44^ led to ‘sneaking around’ to conceal use^34,35,39,44,51,62,63^ or leaving PrEP at friends’ houses.^34^ Being able to discreetly take PrEP (such as transferring the pills into another bottle), increased autonomy and agency while avoiding parental/family gatekeeping and judgement.^45,47,62^ In the United States, Moskowitz et al.^62^ reported that among their samples 62.9% would use PrEP if their parents would not find out, and Owens et al.^63^ found that parental support was significantly associated with greater confidence in PrEP adherence. Within these two studies over 80% of participants reported they would definitely or probably access PrEP if provided for free *and* able to use with discretion (e.g. parents not finding out).^62,63^

Young people in the United States described fears around parents being unsupportive of sexual identities or not being ‘*out*’ to parents as a barrier to access and uptake.^36,48,51,62,63,65^

You’re afraid to even ask your parents, it’s like basically saying, ‘Oh, I want to have gay sex’. And so it’s something that I try and find a way to discreetly do without my parents knowing if possible. And if it’s not possible, it’s probably something I’d just not do. (assigned male at birth; 15 years; U.S.)^36^

Young people from Africa^34,35,37,45^ and the United States^55,62,63^ reported having supportive family members and listing them as clinic contacts^34^ enabled the ‘*freedom*’ to maintain PrEP use by supporting appointments attendance^34,63^ and adherence reminders.^34,35,45^ Parents support of sexual orientation/behaviours enabled young people to feel more comfortable discussing their sexual health needs^34,55,62^ and encouraged them to seek PrEP.^34^ Parental support helped young females in Kenya^37,45^ and Uganda^37^ conceal PrEP use from male partners, helping mitigate the lack of support and HIV risk from partners.

I actually shared [my PrEP use] with my mum. . . .I had a lot of quarrels with my husband and I had to run back to my mum’s house . . . So my mum sat me down and told me that there is no need to keep running away all the time. That I should stay put because it is men’s nature to wander away [have outside partners] when they have cash. She advised me that I should look for a way to protect myself [against HIV] (female; 20 years; Kenya)^45^

#### Do I need my partners permission to use PrEP?

Young women from regions of Africa^35,37,40,44–46,52,56,57^ reported that partners’ accusations of infidelity,^37,^44^–46^ scepticism/misperceptions about partner’s HIV status^35,37,45,46,56^ and traditional power imbalances or normative gender roles^37,44^ influenced perceptions of risk and autonomy to access PrEP. Partners engaging in practices such as concurrent sexual partners^35,37,45^ and polygamy^37^ increased young women’s perception of their HIV risk. Engaging in transactional sex was also found to be associated with higher PrEP use across both quantitative^52,54,56,57^ and qualitative articles.^37^ Difficulties in condom negotiation,^37,44,45^ experiences of physical violence^35,40,46,57^ and reports of partners hiding, confiscating or discarding PrEP pills^35,45^ made young women feel they needed to seek permission from male partners to take PrEP^37^ and/or resort to concealment of use.^35,37,40^ Such experiences of gender-based violence were significantly associated with lower PrEP persistence.^57^

On my side, when I tell my partner that I use PrEP and he does not agree, I will use it secretly. Because these drugs are in bottles and to them, they feel they are ARVs. And because of that, he keeps beating me all the time and because of that I am forced to use them secretly in hiding. (female; 23 years; Kenya)^40^

PrEP use for some female participants was motivated by future plans such as finishing school, having a family, preventing vertical transmission and staying healthy and alive to look after their current children,^37,45,46^ particularly if they felt partners behaviours placed them at risk.^45,46^ Young people in Brazil described PrEP use as unnecessary while being in a monogamous relationship and ‘building trust’ with their partner.^47^ Being able to maintain privacy/discretion when taking PrEP was also a facilitator for use, such as convincing partners it is medication for pregnancy.^45^ Quantitative findings indicated that PrEP use was also significantly associated with having a sexual partner who uses PrEP^52^ with qualitative findings reporting young people describing support from partners also taking PrEP or living with HIV.^35,37,44–47,52,56^

My husband is also taking his medicine [ARVs] at the same time with me, so I have not seen any difficulty [in remembering to take PrEP]. Sometimes if I forget, he reminds me. Sometimes my phone alarm might go off and he reminds that it is time [to take PrEP]. (female; 24 years; Kenya)^45^

### Communities can discourage use – Community stigma can be overcome by supportive peers

Seventeen qualitative articles from across the three contexts (African nations, ^35,37,40,44,46,50,60^ the United States^38,39,41,43,48,51,61,62,65^ and Brazil,^47^) described how community attitudes and stigma prevented PrEP use. Community attitudes and peer disapproval made young people feel discouraged and ‘*embarrassed’* of PrEP use^35,37,39,41,44,48,60^ and fearful of people thinking ‘*you have the disease [HIV]’*^39,65^ or being labelled ‘*dirty*’, a *‘whore’* or as you ‘*sleep around*’^35,37,38,41,43,60,62^ along with fear of judgement, rumours regarding pill recognition,^37,43,44^ and being seen attending clinics.^37,44,50^ One paper from the US described how posting PrEP use on social networking and partner meeting sites was viewed as promoting condom non-use and risky behaviour and was met with negative reactions which led to discontinuation.^48^

And when I say that I take PrEP, she [a friend] thinks that I have sex with everyone under the sun, and that’s why I take PrEP. There’s much prejudice to a person who takes PrEP (male; 17 years; Brazil).^47^

Eight African-based articles^35,37,42,44–46,56,60^ and two from the United States^42,64^ reported on the positive benefits of community, social clubs/groups. Peers provided support through improving PrEP awareness, providing connection and shared experiences, and support for PrEP uptake and continuation.^35,37,44,60,64^ Such support was particularly helpful for participants who had to hide PrEP use from partners.^35,37,56^ Support in the form of a ‘treatment buddy’ (someone to take pills with) or partner provided motivation, encouraged the incorporation of PrEP into daily routine and helped young people to remember to take pills and maintain adherence.^45,60,64^ Having had a sexual partner who used PrEP was significantly associated with PrEP awareness, uptake and use.^52^ Witnessing PrEP use among community peers aided in normalisation, encouraging and empowering young people to use PrEP and attend clinics.^35,44,46^

The best way for them [peers] to use it is when they see me using it. . .Yaahh, they want protection, they need to see me having used it and am alive. Then they will say let’s go boys and we get them [PrEP pills] together (male; 19-21 years; Zimbabwe).^44^

Some young people^39,43,46,47,62,65^ rejected stigma as a barrier, opinions of others were insignificant in their decision-making process. PrEP was viewed as part of their general wellness with one participant describing ‘*If you want to not become sick, you eat oranges. So PrEP is my oranges*.’^65^ Young people claimed personal agency and autonomy, identified as important factors in personal decision-making and use of PrEP.

I feel like you should just be happy that I’m trying to prevent getting HIV instead of worrying about what I’m doing. I feel like as long as I’m taking care of my health, there shouldn’t be a problem. (female; 16 years; United States)^39^

### The healthcare system paradox – The healthcare system itself limits healthcare access

Twenty articles that included nine qualitative articles^36,38,39,41,51,61–63,65^ and five quantitative articles^53,54,58,63^ from the United States, six of the qualitative articles from African nations^35,37,40,44,46,50^ and one qualitative article from Brazil^47^ reported on the impacts healthcare systems and healthcare providers had on access and use of PrEP. These included difficulties navigating healthcare systems, negative experiences, healthcare provider stigma, confidentiality fears and costs associated with accessing medication.

Similar difficulties accessing healthcare were reported across regions of Africa and the United States including proximity to healthcare providers and pharmacies offering PrEP,^37,39,41,44,46,49,50^ clinic closures,^50,51^ long clinic wait times,^44,50^ health centres running out of medications,^44^ a lack of access to affirming healthcare^51^ and reliance on parent transportation.^63^

I live in a conservative rural community and have to drive a long way to a supportive care facility, I just choose to not take PrEP at all right now because of the frequent required visits. (male; 17-24 years; United States)^51^

The need for 3-monthly follow-up appointments was seen as inconvenient, unmanageable, and resulted in scheduling conflicts with school/going away for school, extracurricular activities, and unanticipated events.^36,37,39,49,61,63,65^ Confidentiality fears were shared by young people in African settings around healthcare provider disclosure of PrEP use to parents.^37,44^

Financial barriers were also described across the three settings,^35,38,39,41,43,44,46,47,51,61–63,65^ including medication costs, travel to clinics, healthcare costs, and unstable employment, and living costs. For young people in the United States, further hesitancy occurred through the inability to afford PrEP without the use of parental health insurance, creating fears of raising parental suspicions, and unwanted breaches of confidentiality, especially if young people are not ‘*out*’ to their parents about their sexual identity or behaviours.^38,39,41,51,58,62,63,65^ Unexpected financial strain associated with loss of employment, unstable income, moving and the need to prioritise other essential expenses (e.g. food) also affected PrEP use.^35,38,44,51^

If you know that you are not sick you will say let me go do something else with that money and you overlook your health. . . .if not for paying [for PrEP] then it will be very easy for me. (female 17 years; Uganda).^44^

Providing PrEP at low/no cost, at local clinics/pharmacies and without the need for a prescription was described to make PrEP attainable for young people,^38,44,50,62,63^ while circumventing unwanted parental disclosure.^61^ Having spoken with healthcare providers about PrEP,^54^ and access to one’s own health insurance were significant predictors of greater awareness and access to PrEP.^52,58^ School/college-based health clinics that assured confidentiality and required no parental involvement,^39,^51^^ proximity to affordable local clinics, school-based clinics, PrEP provided through pharmacies and affirming care also made it easier to adhere to PrEP and follow-up appointments.^39,44,46,50,51^

#### HCP practices and attitudes influence care – Negative experiences led to medical mistrust

Across both the United States^36,51,61^ and African settings^37,40,44^ young people described negative experiences with healthcare providers (HCP), including judgement or discrimination of ‘*lifestyle’*, sexual orientation, or sexual partners numbers^36,40,44,51^ and even positive health-seeking behaviours such as regular testing.^36^ Provider-related barriers including lack of HCP knowledge,^36^ unsupportive and non-affirming care,^36,51^ HCPs not providing PrEP to some key populations such as bisexual men^51^ and HCP beliefs (e.g. strong religious beliefs) deterring sexual identity disclosure^36^ were identified as factors that could impact access to PrEP.^36^

There was one time that it wasn’t my primary care doctor. It was a different doctor. . . . and she spoke heavily religiously and was telling me, because she thought I had depression, she was saying going to church would help that. And I felt in my mind if she believes this as a doctor and is recommending this to me, I probably should not tell her about being gay (assigned male at birth; 17 years; United States).^36^

These negative experiences meant that the commonly used words such as ‘*speak to your doctor for more information*’ were a deterrent for young people who did not feel comfortable speaking with their healthcare provider.^36^ Support provided by healthcare providers, such as adherence focused support, PrEP adherence counsellors and regular reminders helped young people maintain adherence.^60,65^ Having trust in their provider and being offered practical advice about side effects, how to discontinue and re-initiate PrEP, and guidance on missed dosages was a facilitator to use.^60^ Providing information enables young people to feel empowered to continue PrEP when faced with challenges such as side effects, stigma, and help educate family and partners to support use.^60^

## Discussion

This review found that factors affecting access, uptake and use of PrEP in young people occur across multiple levels including intrapersonal factors (e.g. knowledge, personal attitudes), interpersonal (parents, partners), community (stigma, community support) and structural (healthcare systems).^13–16^ The synthesis of findings highlights that young people experience commonalities in barriers and facilitators associated with access and uptake of PrEP across low- and high-income countries. While these commonalities are clear, the specific experiences of young people based on their sociocultural contexts should not be overlooked.

At an interpersonal level, key themes identified included limited awareness and knowledge of PrEP uptake and use,^66^ even in contexts where young people are known to be at higher HIV risk.^3,9,13,14,29,67^ As evidenced in studies included in this review and other literature, there are greater disparities in PrEP knowledge linked to younger age groups.^3,66,67^ HIV prevention and PrEP initiatives that include education and media (including television, internet and social media) campaigns aimed at young people have been shown to be effective at not only improving awareness, but also willingness and use of PrEP.^68,69^ There is need for concerted efforts to improve PrEP awareness and knowledge among young people and consider the implementation of such strategies with more targeted approaches to education and promotion of PrEP towards young people to increase awareness and uptake.

Our study highlighted how perceptions of risk vary among young people, but strongly influenced decisions to use PrEP, especially when there is absence of peer representation in PrEP promotion. Currently promotion of PrEP largely relies on people being able to accurately self-identify HIV risk.^70,71^ Accurate risk perception has been defined as a fundamental component in determining engagement with protective behavioural changes, thus inaccurate perceptions can impede engagement with HIV prevention.^4,72–75^ While inaccurate perceptions of risk are known to occur in populations of adults and young people, younger people have a greater likelihood of inaccurately identifying their personal risk.^4,74^ This combined with findings presented in this paper suggest that current promotion and framing PrEP around individual risk of HIV may not be adequately reaching young people.^76^

Our synthesis of research found that some young people articulated PrEP use as empowering, rejecting negative reactions and stigma from others. Prevention strategies that places emphasis on individual behaviour change in young people without consideration of social, cultural and systemic factors (e.g. stigma, financial limitations or poverty, education, healthcare access) that can impact HIV transmission, access to care and create a sense of personal fault that coincides with infection.^77–79^ Representation of young people as stakeholders in the development and design of interventions and messaging is critical to ensure PrEP-related promotion is acceptable and sustainable.^80,81^ Framing of HIV prevention outside of individual behaviour changes is needed, with more focus on promoting community and systems change that support the ability and empowerment of young people to make positive sexual health decisions.^82–84^ The efficacy of PrEP access, uptake and use within some populations is based on targeted messaging (e.g. among MSM in Australia^85,86^), however for some groups this may perpetuate stigmas that PrEP is associated with particular identities or persons engaging in particular ‘behaviours’.^76^ Thus, while targeted messaging is effective, universal health promotion messaging could be effective in reaching boarder populations of young people by removing the emphasis on risk associated only with particular identities or behaviours.^76^

Young people face challenges with HIV-related medication adherence, and those accessing PrEP also have reduced rates of medication adherence, difficulty in adapting to a daily pill-taking regimen and higher rates of discontinuation of use compared to adults.^4,8,87^ This is reflected in our findings along with aversion to taking pills and challenges attending follow-up appointments. Across study settings of the included articles and in other literature,^25,88–91^ the use of support groups, peers/peer-led support and ‘treatment buddies’ were reported to be beneficial in improving medication adherence and improving adherence for follow-up appointments, increasing personal agency, mitigating stigma, reducing isolation, providing side-effect support and increasing open communication about HIV. Expanding adherence support through the use of mobile apps, modifiable or flexible PrEP refill schedules, and the integration of PrEP into other health services could help overcome challenges in clinic attendance and follow-up while also increasing access and convenience for young people regardless of where they live.^92–96^ Few studies described the patterns of PrEP use in young people, highlighting a direction for future research.

Our study highlights the pervasive effects of stigma that surrounds HIV and PrEP and the urgent need to acknowledge and address such stigma. Decision-making in young people is often influenced by their social acceptability, interactions with interpersonal relationships and the broader community.^4,67,97^ Stigma surrounding PrEP use largely overlaps stigma and perceptions around sexual behaviours.^98–100^ Including PrEP use as part of positive sexual health plans and risk-reduction packages targeted towards young people could be an important step towards reducing stigma, increasing education and supporting uptake and continuation of PrEP.^98,101^ The development of personal agency in young people through positive sexual health decision-making and encouraging protective behaviours can also result in lower rates of other sexually transmissible infections and increased HIV testing.^102,103^ Early development of these protective behaviours and ownership over one’s sexual health can promote longevity of these behaviours into adulthood and facilitate greater sexual health communication with partners and engagement with prevention strategies.^103–105^ Our study showed that perceiving PrEP as a positive choice in protecting oneself or family facilitated use and adherence. Therefore, it is important to reframe HIV prevention and PrEP as a positive and proactive sexual health choice.^101–103^

Our findings highlight that it is critical to include families, partners, peers and broader community members in PrEP awareness strategies. Support from family, partners and community, and open discussions surrounding sexual health and HIV have been associated with increased awareness and willingness to use PrEP among young people – as well as reductions in PrEP-related stigma.^97,106–109^ However, peers, partners, family and broader community members can also be gatekeepers or prevent PrEP uptake.^82,110,111^ Therefore, co-designing HIV prevention strategies with young people in collaboration with their broader intergenerational community networks may help facilitate culturally congruent uptake and effective use of PrEP and enhance related health literacy.^80^

This review highlights the importance of considering power discrepancies and gender roles associated with traditional sociocultural normative beliefs and attitudes, particularly among young women. Social support services were reported to help young women navigate these cultural and societal factors, parental and partner influence, gender roles, gender-based violence and inabilities to negotiate condom use – factors known to impact HIV risk and PrEP use in this population.^25,112,113^ Social/peer support, both formal and informal outside of the family and partner network has been found to effectively increase personal agency of young people in relation to their sexual health and communication, increasing protective behaviours, HIV prevention and willingness to access PrEP.^3,99,103–105,114,115^ In contexts where PrEP use is often linked to sexual identity (being MSM), PrEP use can lead to unwanted exposure or being ‘outed’,^116^ such models of peer and social support can provide safe spaces for young people to feel engaged, validated, share experiences and navigate difficulties in maintaining PrEP use.^94,115,117,118^

Healthcare systems can create multiple and cumulative barriers for young people accessing PrEP and HIV services both in high- and low-income contexts accessing PrEP and HIV services.^90,119,120^ Actual or perceived HCP stigma can adversely impact access to HIV prevention/treatment services. Access is further barred by costs associated with clinic attendance and PrEP medications.^99,^120^–122^ Financial barriers can be exacerbated for young people who commonly fall into the lowest income bracket.^123^ Financial dependence on parents or guardians can create barriers through concerns around confidentiality.^3,^124^–126^ The provision of PrEP at no or subsidised costs for young people has been linked to an increased willingness to use PrEP,^69^ and is effective in national uptake of PrEP.^69,127–129^ Young people in our included studies cited perceived costs as barriers to uptake, it is important that young people are made aware of programmes that can offer PrEP for free to support access and uptake. For example, young people in the United States can access *Ready, Set, PrEP*^130^ a programme offering free PrEP to people without health insurance in the United States. Support is needed for HCP to overcome barriers to the provision of PrEP to young people such as lack of awareness, reduced sense of need in young people and lack comfortability in initiating sexual health and HIV conversations.^3,131–134^ However, reducing medication and health service cost alone is not sufficient. Our review emphasises the need to consider travel costs and other related cost barriers such as living expenses and unstable or loss of employment. HCP need to be aware of the risk and stigma facing young people trying to access PrEP and their duty of care to provide equitable access to supportive non-judgemental healthcare that ensures confidentiality.^99,122^ Additionally, expanding PrEP delivery through non-traditional models of care (e.g. through nurse-led and pharmacy-led PrEP) can be effective in overcoming some of these challenges for young people by improving accessibility, anonymity and autonomy.^135,136^

The findings highlight how young people experience intersecting barriers and facilitators to PrEP uptake, use and adherence. Clearly, future research and practice need to be collaborative with young people to design strategies to overcome barriers and facilitate access. Research and practice must also engage with the broader holistic sociocultural context of young people to create meaningful change. Summarised in Table 3 are key recommendations for practice, research, and policy based on our findings to guide collaborative and holistic health promotion strategies and interventions with young people.

**Table 3.** Key recommendations for practice, research and policy to support PrEP access, uptake and use in young people.

1. Include young people as stakeholders in the development and design of interventions and messaging to ensure promotion is acceptable, culturally tailored, sustainable and accessible 2. Provide targeted and universal health promotion messaging to reach boarder populations of young people 3. Expanding adherence support through the use of mobile apps or clinic text message reminders 4. Including PrEP as part of positive sexual health plans and risk-reduction packages targeted towards young people 5. Reframe HIV prevention and PrEP as a positive and proactive sexual health choice to reduce stigma 6. Co-designing HIV prevention strategies with young people in collaboration with their broader intergenerational community networks to facilitate culturally congruent uptake and use of PrEP and enhance related health literacy 7. Develop accessible peer and social support to provide safe spaces for young people to feel engaged, validated, provide adherence support and empowerment to navigate difficulties in maintaining PrEP use 8. The provision of PrEP at no or subsidised costs for young people and ensure young people are aware of initiatives to provide financial assistance and access to PrEP 9. Education and support for healthcare providers to improve information, education and provision of PrEP to young people 10. Expand PrEP delivery through non-traditional models of care (e.g. through nurse-led and pharmacy-led PrEP, or flexible PrEP refill schedules) to improve accessibility, anonymity and autonomy

PrEP, pre-exposure prophylaxis.

## Strengths and limitations

This study provides insight into the differing impacts that young people face in access and use of HIV prevention, however the inclusion of articles from only African nations, the United States and Brazil, limits the generalisability of our findings within other geographical and sociocultural contexts. The articles included in this review did not report other factors around the age of consent or laws pertaining to healthcare or medications, which could further impact access to services and PrEP.^2,126^

## Conclusion

There is a need to move beyond prevention efforts that address only individual-level barriers to PrEP for young people. PrEP health promotion strategies and services need to consider the impacts of social, cultural and systemic structures on HIV transmission and prevention in young people. There is a need for a multiprong approach, supported by appropriate legislation, policy, and systems, designed to increase PrEP awareness across all ages, develop supportive social networks within interpersonal networks and the wider community, and improve PrEP service delivery and access. Improved access to affordable age appropriate culturally congruent, affirming PrEP services is imperative to support the needs of young people. Without considerations for these wider implications to access, uptake and use of HIV prevention and PrEP, global targets towards the elimination of HIV transmission by 2030 will remain out of reach.

## Supplemental Material

sj-docx-1-tai-10.1177_20499361241303415 – Supplemental material for Pre-exposure prophylaxis access, uptake and usage by young people: a systematic review of barriers and facilitators Supplemental material, sj-docx-1-tai-10.1177_20499361241303415 for Pre-exposure prophylaxis access, uptake and usage by young people: a systematic review of barriers and facilitators by Sarah Warzywoda, James A. Fowler, Amalie Dyda, Lisa Fitzgerald, Amy B. Mullens and Judith A. Dean in Therapeutic Advances in Infectious Disease

sj-docx-2-tai-10.1177_20499361241303415 – Supplemental material for Pre-exposure prophylaxis access, uptake and usage by young people: a systematic review of barriers and facilitators Supplemental material, sj-docx-2-tai-10.1177_20499361241303415 for Pre-exposure prophylaxis access, uptake and usage by young people: a systematic review of barriers and facilitators by Sarah Warzywoda, James A. Fowler, Amalie Dyda, Lisa Fitzgerald, Amy B. Mullens and Judith A. Dean in Therapeutic Advances in Infectious Disease

## Declarations

**Ethics approval and consent to participate:** Ethical approval for this study was obtained from The University of Queensland (2022/HE000560). Informed consent was not obtained for this study.

**Consent for publication:** Not applicable.

**Availability of data and materials:** As this is a systematic review, the authors do not hold access to individual study’s data beyond what is publicly available in the published article.

## References

[1] Hegde A Chandran S Pattnaik JI. Understanding adolescent sexuality: a developmental perspective. J Psychosexual Health 2022; 4(4): 237–242.

[2] Taggart T Bond KT Ritchwood TD , et al. Getting youth PrEPared: adolescent consent laws and implications for the availability of PrEP among youth in countries outside of the United States. J Int AIDS Soc 2019; 22(7): e25363. 10.1002/jia2.25363 PMC6672744 31369211

[3] Yusuf H Fields E Arrington-Sanders R , et al. HIV Preexposure prophylaxis among adolescents in the US: a review. JAMA Pediatr 2020; 174(11): 1102–1108. 32391878 10.1001/jamapediatrics.2020.0824

[4] Haberer JE Mugo N Baeten JM , et al. PrEP as a lifestyle and investment for adolescent girls and young women in Sub-Saharan Africa. J Int Assoc Provid AIDS Care 2019; 18: 2325958219831011. 10.1177/2325958219831011 PMC6748528 30776954

[5] Kahle EM Hughes JP Lingappa JR , et al. An empiric risk scoring tool for identifying high-risk heterosexual HIV-1-serodiscordant couples for targeted HIV-1 prevention. J Acquir Immune Defic Syndr 2013; 62(3): 339–347. 23187945 10.1097/QAI.0b013e31827e622d PMC3620695

[6] Hammoud MA Vaccher S Jin F , et al. HIV Pre-exposure prophylaxis (PrEP) uptake among gay and bisexual men in Australia and factors associated with the nonuse of PrEP among eligible men: results from a prospective cohort study. J Acquir Immune Defic Syndr 2019; 81(3): e73–e84. 10.1097/QAI.0000000000002047 30973548

[7] Hosek S Celum C Wilson CM , et al. Preventing HIV among adolescents with oral PrEP: observations and challenges in the United States and South Africa. J Int AIDS Soc 2016; 19(7S6): 21107. 27760684 10.7448/IAS.19.7.21107 PMC5071778

[8] Huebner DM Mustanski B. Navigating the long road forward for maximizing PrEP impact among adolescent men who have sex with men. Arch Sex Behav 2020;49(1):211–216. 31667642 10.1007/s10508-019-1454-1 PMC7665846

[9] WHO. HIV: World Health Organization, https://www.who.int/news-room/fact-sheets/detail/hiv-aids (2022, accessed 15 August 2023).

[10] UNICEF. Adolescent HIV prevention. https://data.unicef.org/topic/hivaids/adolescents-young-people/ (2023, accessed 15 August 2023).

[11] As world marks arrival of 8 Billionth citizen, the largest ever generation of youth call for change [press release]. The Partnership for Maternal, Newborn & Child Health (PMNCH), https://pmnch.who.int/news-and-events/news/item/18-11-2022-as-world-marks-arrival-of-8-billionth-citizen-the-largest-ever-generation-of-youth-call-for-change (2022, accessed 20 August 2023).

[12] World Bank. World development indicators database. World Bank, https://datacatalog.worldbank.org/search/dataset/0037712 (2023, accessed 20 August 2023).

[13] Centers for Disease Control and Prevention (CDC). HIV surveillance report. U.S. Department of Health & Human Services, https://www.cdc.gov/hiv/library/reports/hiv-surveillance/vol-33/index.html (2022, accessed 15 August 2023).

[14] Lyons SJ Johnson AS Hu X , et al. Monitoring selected national HIV prevention and care objectives by using HIV surveillance data : United States and 6 dependent areas, 2020. https://stacks.cdc.gov/view/cdc/117803 (2022, accessed 15 August 2023).

[15] UNAIDS. Young people and HIV. Joint United Nations Programme on HIV/AIDS (UNAIDS), https://www.unaids.org/sites/default/files/media_asset/young-people-and-hiv_en.pdf (2021, accessed 21 August 2023).

[16] UNAIDS. Fact Sheet 2022: Global HIV statistics, https://www.unaids.org/sites/default/files/media_asset/UNAIDS_FactSheet_en.pdf (2022, accessed 9 September 2023.

[17] Ramraj T Chirinda W Jonas K , et al. Service delivery models that promote linkages to PrEP for adolescent girls and young women and men in sub-Saharan Africa: a scoping review. BMJ Open 2023; 13(3): e061503. 10.1136/bmjopen-2022-061503 PMC10069497 36972966

[18] UNAIDS. Women, adolescent girls and the HIV response. Joint United Nations Programme on HIV/AIDS (UNAIDS), 2020.

[19] Philbin MM Parker CM Parker RG , et al. The promise of pre-exposure prophylaxis for black men who have sex with men: an ecological approach to attitudes, beliefs, and barriers. AIDS Patient Care STDs 2016; 30(6): 282–290. 27220036 10.1089/apc.2016.0037 PMC4913505

[20] Bronfenbrenner U. Toward an experimental ecology of human development. Am Psychol 1977; 32(7): 513–531.

[21] Hodges-Mameletzis I Dalal S Msimanga-Radebe B , et al. Going global: the adoption of the World Health Organization’s enabling recommendation on oral pre-exposure prophylaxis for HIV. Sex Health 2018;15(6):489–500. 30496718 10.1071/SH18125

[22] U.S. Food and Drug Administration Approves Expanded Indication for Truvada® (Emtricitabine and Tenofovir Disoproxil Fumarate) for Reducing the Risk of Acquiring HIV-1 in Adolescents [press release]. Foster City, California: Gilead Sciences, Inc. 2018.

[23] Kirby T. PrEP finally approved on NHS in England. Lancet 2020; 395(10229): 1025. 32222189 10.1016/S0140-6736(20)30720-0

[24] AIDS Committee of Toronto (ACT). HIVnow Times have changed: paying for PrEP in Ontario, https://www.get-prep.com (2017, accessed 7 September 2023).

[25] Bärnighausen K Geldsetzer P Matse S , et al. Qualitative accounts of PrEP discontinuation from the general population in Eswatini. Cult Health Sex 2021; 23(9): 1198–1214. 32633617 10.1080/13691058.2020.1770333

[26] AIDES. PrEP instructions for use, https://www.aides.org/prep (2022, accessed 7 September 2023).

[27] Munn Z Peters MDJ Stern C , et al. Systematic review or scoping review? Guidance for authors when choosing between a systematic or scoping review approach. BMC Med Res Methodol 2018; 18(1): 143. 30453902 10.1186/s12874-018-0611-x PMC6245623

[28] UNAIDS. Understanding fast-track: accelerating action to end the AIDS epidemic by 2030. Geneva, Switzerland: Joint United Nations Programme on HIV/AIDS, 2015.

[29] UNAIDS. Ending the AIDS epidemic for adolescents, with adolescents. Geneva, Switzerland: Joint United Nations Programme on HIV/AIDS, 2016.

[30] WHO. Adolescent Health: World Health Organization, https://www.who.int/southeastasia/health-topics/adolescent-health (2021, accessed 15 August 2023).

[31] The EndNote Team. EndNote. 20 ed. Philadelphia, PA: Clarivate, 2013.

[32] Popay J Roberts H Sowden A , et al. Guidance on the conduct of narrative synthesis in systematic reviews, https://citeseerx.ist.psu.edu/document?repid=rep1&type=pdf&doi=ed8b23836338f6fdea0cc55e161b0fc5805f9e27 (2006, accessed 7 September 2023).

[33] Hong Q Pluye P Fàbregues S , et al. Mixed methods appraisal tool (MMAT), version 2018. Canada, http://mixedmethodsappraisaltoolpublic.pbworks.com/w/file/fetch/127916259/MMAT_2018_criteria-manual_2018-08-01_ENG.pdf (2018, accessed 13 September 2024).

[34] Atujuna M Montgomery ET Hartmann M , et al. The role of families in adolescent and young adults’ PrEP use. AIDS Behav 2022; 26(5): 1618–1632. 34716835 10.1007/s10461-021-03514-3 PMC12968712

[35] Baron D Scorgie F Ramskin L , et al. You talk about problems until you feel free: South African adolescent girls’ and young women’s narratives on the value of HIV prevention peer support clubs. BMC Public Health 2020; 20(1): 1016. 10.1186/s12889-020-09115-4 PMC7320560 32590969

[36] Birnholtz J Kraus A Schnuer S , et al. ‘Oh, I don’t really want to bother with that:’ gay and bisexual young men’s perceptions of barriers to PrEP information and uptake. Cult Health Sex 2022; 24(11): 1548–1562. 34524938 10.1080/13691058.2021.1975825 PMC8920939

[37] Camlin CS Koss CA Getahun M , et al. Understanding demand for PrEP and early experiences of PrEP use among young adults in rural Kenya and Uganda: a qualitative study. AIDS Behav 2020; 24(7): 2149–2162. 31955361 10.1007/s10461-020-02780-x PMC7909847

[38] Crooks N Singer RB Smith A , et al. Barriers to PrEP uptake among Black female adolescents and emerging adults. Prev Med Rep 2022; 31: 102062. 36467542 10.1016/j.pmedr.2022.102062 PMC9712981

[39] Gailloud L Gonzalez-Argoti T Philip S , et al. ‘How come they don’t talk about it in school?’ Identifying adolescent barriers to PrEP use. Health Educ Res 2022; 36(5): 505–517. 34467401 10.1093/her/cyab030 PMC8793170

[40] Hartmann M Otticha S Agot K , et al. Tu’Washindi na PrEP: working with young women and service providers to design an intervention for PrEP uptake and adherence in the context of gender-based violence. AIDS Educ Prev 2021; 33(2): 103–119. 33821679 10.1521/aeap.2021.33.2.103 PMC8384060

[41] Hess KM Crawford J Eanes A , et al. Reasons why young men who have sex with men report not using HIV pre-exposure prophylaxis: perceptions of burden, need, and safety. AIDS Patient Care STDS 2019; 33(10): 449–454. 31584856 10.1089/apc.2019.0150 PMC6785168

[42] Marsh KJ Rothenberger M. A young Black MSM on PrEP is lost to follow-up and acquires HIV infection: a case to call for improved strategies to support youth adherence and engagement in HIV prevention. J Int Assoc Provid AIDS Care 2019; 18: 2325958219853834. doi:10.1177/2325958219853834. 10.1177/2325958219853834 PMC6748537 31159635

[43] McKetchnie SM White B Fontenot H , et al. Perspectives of young men who have sex with men on PrEP adherence and peer navigation: a qualitative study. Arch Sex Behav 2023; 52(5): 2037–2049. 36940046 10.1007/s10508-023-02579-6 PMC10330054

[44] Muhumuza R Ssemata AS Kakande A , et al. Exploring perceived barriers and facilitators of PrEP uptake among young people in Uganda, Zimbabwe, and South Africa. Arch Sex Behav 2021; 50(4): 1729–1742. 33954824 10.1007/s10508-020-01880-y PMC8213546

[bibr45-20499361241303415] Pintye J O’Malley G Kinuthia J , et al. Influences on early discontinuation and persistence of daily oral PrEP use among Kenyan adolescent girls and young women: a qualitative evaluation from a PrEP implementation program. J Acquir Immune Defic Syndr 1999; 2021; 86(4): e83–e89. 10.1097/QAI.0000000000002587 PMC8935942 33273211

[46] Rogers Z Pintye J Kinuthia J , et al. Key influences on the decision to initiate PrEP among adolescent girls and young women within routine maternal child health and family planning clinics in Western Kenya. AIDS Care 2021; 34(3): 363–370. 10.1080/09540121.2021.1981217 PMC8934309 34543077

[47] Santos LAD Unsain RF Brasil SA , et al. PrEP perception and experiences of adolescent and young gay and bisexual men: an intersectional analysis. Cad Saude Publica 2023; 39 (Suppl. 1): e00134421. 10.1590/0102-311XEN134421 36995863

[48] Shorrock F Alvarenga A Hailey-Fair K , et al. Dismantling barriers and transforming the future of pre-exposure prophylaxis uptake in young Black and Latinx sexual minority men and transgender women. AIDS Patient Care STDS 2022; 36(5): 194–203. 35507322 10.1089/apc.2021.0222 PMC9125574

[49] Tapsoba JdD Cover J Obong’o C , et al. Continued attendance in a PrEP program despite low adherence and non-protective drug levels among adolescent girls and young women in Kenya: results from a prospective cohort study. PLoS Med 2022; 19(9): e1004097. 10.1371/journal.pmed.1004097 PMC9521917 36095005

[50] Vera M Bukusi E Achieng P , et al. Pharmacies are everywhere, and you can get it at any time: Experiences with pharmacy-based PrEP delivery among adolescent girls and young women in Kisumu, Kenya. J Int Assoc Provid AIDS Care 2023; 22: 23259582231215882. doi:10.1177/23259582231215882. 10.1177/23259582231215882 PMC10676062 37997351

[51] Zapata JP Dang M Quinn KG , et al. COVID-19-related disruptions to HIV testing and prevention among young sexual minority men 17-24 years old: a qualitative study using synchronous online focus groups, April-September 2020. Arch Sex Behav 2022; 51(1): 303–314. 34773214 10.1007/s10508-021-02166-7 PMC8589091

[52] Bonett S Dowshen N Bauermeister J , et al. Characterizing the PrEP continuum for Black and Latinx sexual and gender minority youth. AIDS Behav 2022; 26(4): 1211–1221. 34546472 10.1007/s10461-021-03476-6 PMC8934745

[53] Hong C Horvath KJ Stephenson R , et al. PrEP use and persistence among young sexual minority men 17-24 years old during the COVID-19 pandemic. AIDS Behav 2022; 26(3): 631–638. 34387777 10.1007/s10461-021-03423-5 PMC8361406

[54] Macapagal K Kraus A Korpak AK , et al. PrEP awareness, uptake, barriers, and correlates among adolescents assigned male at birth who have sex with males in the U.S. Arch Sex Behav 2020; 49(1): 113–124. 31602584 10.1007/s10508-019-1429-2 PMC7263631

[55] Moskowitz DA Moran KO Matson M , et al. The PrEP cascade in a national cohort of adolescent men who have sex with men. J Acquir Immune Defic Syndr 2021; 86(5): 536–543. 33399311 10.1097/QAI.0000000000002613

[56] Sila J Larsen AM Kinuthia J , et al. High awareness, yet low uptake, of pre-exposure prophylaxis among adolescent girls and young women within family planning clinics in Kenya. AIDS Patient Care STDs 2020; 34(8): 336–343. 32757980 10.1089/apc.2020.0037 PMC7415219

[57] Tapsoba JdD Zangeneh SZ Appelmans E , et al. Persistence of oral pre-exposure prophylaxis (PrEP) among adolescent girls and young women initiating PrEP for HIV prevention in Kenya. AIDS Care 2021; 33(6): 712–720. 32951437 10.1080/09540121.2020.1822505 PMC7981281

[58] Whitfield THF Parsons JT Rendina HJ. Rates of pre-exposure prophylaxis use and discontinuation among a large U.S. national sample of sexual minority men and adolescents. Arch Sex Behav 2020; 49(1): 103–112. 31845148 10.1007/s10508-019-01602-z PMC7028359

[59] Zeballos D Magno L Soares F , et al. Oral pre-exposure prophylaxis for HIV discontinuation in a large cohort of adolescent men who have sex with men and transgender women in Brazil. J Adolesc Health 2023; 73(6): S43–S49. 10.1016/j.jadohealth.2023.08.005 37953008

[60] Barnabee G O’Bryan G Ndeikemona L , et al. Improving HIV pre-exposure prophylaxis persistence among adolescent girls and young women: Insights from a mixed-methods evaluation of community, hybrid, and facility service delivery models in Namibia. Front Reprod Health 2022; 4: 1048702. 36545490 10.3389/frph.2022.1048702 PMC9760915

[61] Horvath KJ Todd K Arayasirikul S , et al. Underutilization of pre-exposure prophylaxis services among transgender and nonbinary youth: findings from project Moxie and TechStep. Transgend Health 2019; 4(1): 217–221. 31592151 10.1089/trgh.2019.0027 PMC6778317

[62] Moskowitz DA Macapagal K Mongrella M , et al. What if my dad finds out!? Assessing adolescent men who have sex with men’s perceptions about parents as barriers to PrEP uptake. AIDS Behav 2020; 24(9): 2703–2719. 32157491 10.1007/s10461-020-02827-z PMC7462124

[63] Owens C Moran K Mongrella M , et al. “It’s very inconvenient for me”: a mixed-method study assessing barriers and facilitators of adolescent sexual minority males attending PrEP follow-up appointments. AIDS Behav 2021; 26(1): 21–34. 34081237 10.1007/s10461-021-03313-w PMC8910568

[64] Wood S Dowshen N Bauermeister JA , et al. Social support networks among young men and transgender women of color receiving HIV pre-exposure prophylaxis. J Adolesc Health 2020; 66(3): 268–274. 31672523 10.1016/j.jadohealth.2019.08.014 PMC7007865

[65] Wood S Gross R Shea JA , et al. Barriers and facilitators of PrEP adherence for young men and transgender women of color. AIDS Behav 2019; 23(10): 2719–2729. 30993479 10.1007/s10461-019-02502-y PMC6790163

[66] Fields EL Thornton N Long A , et al. Young black MSM’s exposures to and discussions about PrEP while navigating geosocial networking apps. J LGBT Youth 2021; 18(1): 23–39. 34109014 10.1080/19361653.2019.1700205 PMC8186480

[67] Shamu S Shamu P Khupakonke S , et al. Pre-exposure prophylaxis (PrEP) awareness, attitudes and uptake willingness among young people: gender differences and associated factors in two South African districts. Glob Health Action 2021; 14(1): 1886455. 33606603 10.1080/16549716.2021.1886455 PMC7899653

[68] Birdthistle I Mulwa S Sarrassat S , et al. Effects of a multimedia campaign on HIV self-testing and PrEP outcomes among young people in South Africa: a mixed-methods impact evaluation of ‘MTV Shuga Down South’. BMJ Glob Health 2022;7(4): e007641. 10.1136/bmjgh-2021-007641 PMC8977807 35365480

[69] Sophus AI Mitchell JW. A review of approaches used to increase awareness of pre-exposure prophylaxis (PrEP) in the United States. AIDS Behav 2019; 23(7): 1749–1770. 30306434 10.1007/s10461-018-2305-0

[70] Centers for Disease Control and Prevention. PrEP 101 Pocket Guide. Centers for Disease Control and Prevention, https://www.cdc.gov/hiv/pdf/library/pocket-guides/cdc-hiv-pocket-guide-prep.pdf (2022, accessed 20 September 2023).

[71] Centers for Disease Control and Prevention. Lets stop HIV together https://www.cdc.gov/stophivtogether/index.html (2022, accessed 7 September 2023).

[72] Di Ciaccio M Sagaon-Teyssier L Protière C , et al. Impact of HIV risk perception on both pre-exposure prophylaxis and condom use. J Health Psychol 2019; 26(10): 1575–1586. 31647330 10.1177/1359105319883927

[73] Li YH Mgbere O Abughosh S , et al. Assessment of sexually transmitted disease/HIV risk among young African Americans: comparison of self-perceived and epidemiological risks utilizing ecodevelopmental theory. HIV AIDS 2019; 11: 31–44. 10.2147/HIV.S189482 PMC6388744 30863188

[74] Ndugwa Kabwama S Berg-Beckhoff G . The association between HIV/AIDS-related knowledge and perception of risk for infection: a systematic review. Perspect Public Health 2015; 135(6): 299–308. 26253643 10.1177/1757913915595831

[75] Albarracín D Johnson BT Fishbein M , et al. Theories of reasoned action and planned behavior as models of condom use: a meta-analysis. Psychol Bull 2001; 127(1): 142–161. 11271752 10.1037/0033-2909.127.1.142 PMC4780418

[76] Rivet Amico K Bekker L-G . Global PrEP roll-out: recommendations for programmatic success. Lancet HIV 2019; 6(2): e137–e140. 10.1016/S2352-3018(19)30002-5 30660592

[77] Thomas K. A better life for some: the lovelife campaign and HIV/AIDS in South Africa. Agenda 2004; 18(62): 29–35.

[78] Anugwom E Anugwom K. Socio-cultural factors in the access of women to HIV/AIDS prevention and treatment services in South-southern Nigeria. Iran J Public Health 2016; 45(6): 754–760. 27648418 PMC5026830

[79] Levison JH Levinson JK Alegría M. A critical review and commentary on the challenges in engaging HIV-infected Latinos in the continuum of HIV care. AIDS Behav 2018; 22(8): 2500–2512. 29948334 10.1007/s10461-018-2187-1 PMC6085100

[80] Mackworth-Young C Dringus S Dauya E , et al. Putting youth at the centre: co-design of a community-based intervention to improve HIV outcomes among youth in Zimbabwe. Wellcome Open Res 2022; 7: 53. 38264344 10.12688/wellcomeopenres.17531.2 PMC10804048

[81] Mukherjee TI Zerbe A Falcao J , et al. Human-centered design for public health innovation: codesigning a multicomponent intervention to support youth across the HIV Care Continuum in Mozambique. Glob Health Sci Pract 2022;10(2): e2100664. 10.9745/GHSP-D-21-00664 PMC9053144 35487546

[82] Lirios A Mullens AB Daken K , et al. Sexual and reproductive health literacy of culturally and linguistically diverse young people in Australia: a systematic review. Cult Health Sex 2024; 26(6): 790–807. 37755697 10.1080/13691058.2023.2256376

[83] Leininger M. Gadsup of Papua New Guinea revisited: a three decade view. J Transcult Nurs 1993; 5(1): 21–30. 8217010 10.1177/104365969300500104

[84] McFarland MR Wehbe-Alamah HB. Leininger’s theory of culture care diversity and universality: an overview with a historical retrospective and a view toward the future. J Transcult Nurs 2019; 30(6): 540–557. 31409201 10.1177/1043659619867134

[85] King J McManus H Kwon A , et al. HIV, viral hepatitis and sexually transmissible infections in Australia Annual Surveilance Report 2022. The Kirby Institute, UNSW Sydney, Sydney, Australia, 2022.

[86] ACON. Ending HIV 2022, https://endinghiv.org.au/tribes/young-gay-men/ (2022, accessed 13 September 2023).

[87] Morgan E Ryan DT Newcomb ME , et al. High rate of discontinuation may diminish PrEP coverage among young men who have sex with men. AIDS Behav 2018; 22(11): 3645–3648. 29728950 10.1007/s10461-018-2125-2 PMC6204096

[88] Lazuardi E Newman CE Anintya I , et al. Increasing HIV treatment access, uptake and use among men who have sex with men in urban Indonesia: evidence from a qualitative study in three cities. Health Policy Plan 2020; 35(1): 16–25. 31625559 10.1093/heapol/czz128

[89] Liamputtong P Haritavorn N Kiatying-Angsulee N. HIV and AIDS, stigma and AIDS support groups: perspectives from women living with HIV and AIDS in central Thailand. Soc Sci Med 2009; 69(6): 862–868. 19539417 10.1016/j.socscimed.2009.05.040

[90] Woollett N Pahad S Black V. “We need our own clinics”: adolescents’ living with HIV recommendations for a responsive health system. PLoS One 2021; 16(7): e0253984. 10.1371/journal.pone.0253984 PMC8248739 34197529

[91] Alcon S Ahmed B Sloane D , et al. Interventions to improve medication adherence in adolescents with HIV: a systematic review and meta-analysis. J Investig Med 2020; 68(7): 1217. 10.1136/jim-2020-001295 32699066

[92] Abdulrahman SA Rampal L Ibrahim F , et al. Mobile phone reminders and peer counseling improve adherence and treatment outcomes of patients on ART in Malaysia: a randomized clinical trial. PLoS One 2017; 12(5): e0177698. 10.1371/journal.pone.0177698 PMC5433794 28520768

[93] Fuchs JD Stojanovski K Vittinghoff E , et al. A mobile health strategy to support adherence to antiretroviral preexposure prophylaxis. AIDS Patient Care STDs 2018; 32(3): 104-11. 10.1089/apc.2017.0255 PMC5865612 29565183

[94] Irungu E Khoza N Velloza J. Multi-level interventions to promote oral pre-exposure prophylaxis use among adolescent girls and young women: a review of recent research. Curr HIV/AIDS Rep 2021; 18(6): 490–499. 34719745 10.1007/s11904-021-00576-9 PMC8557703

[95] Aicken CRH Fuller SS Sutcliffe LJ , et al. Young people’s perceptions of smartphone-enabled self-testing and online care for sexually transmitted infections: qualitative interview study. BMC Public Health 2016; 16(1): 974. 27624633 10.1186/s12889-016-3648-y PMC5022229

[96] Liu A Coleman K Bojan K , et al. Developing a mobile app (LYNX) to support linkage to HIV/sexually transmitted infection testing and pre-exposure prophylaxis for young men who have sex with men: protocol for a randomized controlled trial. JMIR Res Protoc 2019; 8(1): e10659. 10.2196/10659 PMC6367663 30681964

[97] Giovenco D Gill K Fynn L , et al. Experiences of oral pre-exposure prophylaxis (PrEP) use disclosure among South African adolescent girls and young women and its perceived impact on adherence. PLoS One 2021; 16(3): e0248307. 10.1371/journal.pone.0248307 PMC7935254 33667264

[98] Golub SA. PrEP stigma: implicit and explicit drivers of disparity. Curr HIV/AIDS Rep 2018; 15(2): 190–197. 29460223 10.1007/s11904-018-0385-0 PMC5884731

[99] Yoshioka E Giovenco D Kuo C , et al. “I’m doing this test so I can benefit from PrEP”: exploring HIV testing barriers/facilitators and implementation of pre-exposure prophylaxis among South African adolescents. Afr J AIDS Res 2020; 19(2): 101–108. 32326813 10.2989/16085906.2020.1743726 PMC8006570

[100] Radcliffe J Doty N Hawkins LA , et al. Stigma and sexual health risk in HIV-positive african American young men who have sex with men. AIDS Patient Care STDs 2010; 24(8): 493–499. 20673080 10.1089/apc.2010.0020 PMC4932787

[101] Taylor SW Psaros C Pantalone DW , et al. “Life-steps” for PrEP adherence: demonstration of a CBT-based intervention to increase adherence to preexposure prophylaxis (PrEP) medication among sexual-minority men at high risk for HIV acquisition. Cogn Behav Pract 2017; 24(1): 38–49. 28392673 10.1016/j.cbpra.2016.02.004 PMC5381825

[102] Keene L Boyd D . Ending the epidemic: assessing sexual health communication, personal agency, and HIV stigma among Black and Latino youth in the U.S. Int J Environ Res Public Health 2021; 18(12): 6319. 34207968 10.3390/ijerph18126319 PMC8296133

[103] Boyd DT Waller B Quinn CR. Reimaging an AIDS free generation: examining youth and young adults’ personal agency and its association with HIV testing. Prev Med Rep 2021; 22: 101335. 33680722 10.1016/j.pmedr.2021.101335 PMC7930588

[104] Widman L Choukas-Bradley S , et al. Sexual communication between early adolescents and their dating partners, parents, and best friends. J Sex Res 2014; 51(7): 731–741. 24354655 10.1080/00224499.2013.843148 PMC4063897

[105] Boyd D Lea CH Gilbert KL , et al. Sexual health conversations: predicting the odds of HIV testing among black youth and young adults. Child Youth Serv Rev 2018; 90: 134–140.

[106] Ajayi AI Mudefi E Yusuf MS , et al. Low awareness and use of pre-exposure prophylaxis among adolescents and young adults in high HIV and sexual violence prevalence settings. Medicine 2019; 98(43): e17716. 10.1097/MD.0000000000017716 PMC6824740 31651904

[107] Boyd DT Abubakari GM Turner D , et al. The influence of family bonding, support, engagement in healthcare, on PrEP stigma among young Black and Latino men who have sex with men: a path analysis. Children 2022;9(3): 330. 35327703 10.3390/children9030330 PMC8947403

[108] Thoma BC Huebner DM. Brief report: HIV pre-exposure prophylaxis engagement among adolescent men who have sex with men: the role of parent-adolescent communication about sex. J Acquir Immune Defic Syndr 2018; 79(4): 453–457. 30371531 10.1097/QAI.0000000000001837 PMC6211193

[109] Mullens AB Kelly J Debattista J , et al. Exploring HIV risks, testing and prevention among sub-Saharan African community members in Australia. Int J Equity Health 2018; 17(1): 62. 29784050 10.1186/s12939-018-0772-6 PMC5963033

[110] Dean J Mitchell M Stewart D , et al. Intergenerational variation in sexual health attitudes and beliefs among Sudanese refugee communities in Australia. Cult Health Sex 2017; 19(1): 17–31. 27268405 10.1080/13691058.2016.1184316

[111] Istiko SN Remata S Ndayizeye A , et al. Developing critical HIV health literacy: insights from interviews with priority migrant communities in Queensland, Australia. Cult Health Sex 2024; 26(7): 936–951 37950430 10.1080/13691058.2023.2265960

[112] Murewanhema G Musuka G Moyo P , et al. HIV and adolescent girls and young women in sub-Saharan Africa: a call for expedited action to reduce new infections. IJID Reg 2022; 5: 30–32. 36147901 10.1016/j.ijregi.2022.08.009 PMC9485902

[113] Velloza J Khoza N Scorgie F , et al. The influence of HIV-related stigma on PrEP disclosure and adherence among adolescent girls and young women in HPTN 082: a qualitative study. J Int AIDS Soc 2020; 23(3): e25463. 10.1002/jia2.25463 PMC7060297 32144874

[114] Boyd DT Waller B Quinn CR. Understanding of personal agency among youth to curtail HIV rates. Children and Youth Serv Rev 2020; 116: 105179. 36778097 10.1016/j.childyouth.2020.105179 PMC9912712

[115] Dacus J-d Voisin DR Barker J. “Proud I am negative”: maintaining HIV-seronegativity among Black MSM in New York City. Men and Masculinities 2017; 21(2): 276–290.

[116] Fisher CB Fried AL Ibrahim Puri L , et al. “Free testing and PrEP without outing myself to parents:” motivation to participate in oral and injectable PrEP clinical trials among adolescent men who have sex with men. PLoS One 2018; 13(7): e0200560. 10.1371/journal.pone.0200560 PMC6059443 30044845

[117] Ortiz E Scanlon B Mullens A , et al. Effectiveness of interventions for hepatitis B and C: a systematic review of vaccination, screening, health promotion and linkage to care within higher income countries. J Commun Health 2020; 45(1): 201–218. 10.1007/s10900-019-00699-6 31332639

[118] Hollingdrake O Lui C-W Dean JA , et al. “They’re my go-to people”: a qualitative study of support networks for people recently diagnosed with HIV in Queensland, Australia. J Assoc Nurses AIDS Care 2022; 33(6): 624–637. 35878321 10.1097/JNC.0000000000000351

[119] Kay ES Pinto RM. Is insurance a barrier to HIV preexposure prophylaxis? Clarifying the issue. Am J Public Health 2020; 110(1): 61–64. 31725314 10.2105/AJPH.2019.305389 PMC6893325

[120] Lazarou M Fitzgerald L Warner M , et al. Australian interdisciplinary healthcare providers’ perspectives on the effects of broader pre-exposure prophylaxis (PrEP) access on uptake and service delivery: a qualitative study. Sex Health 2020; 17(6): 485–492. 33292927 10.1071/SH20156

[bibr121-20499361241303415] Pleuhs B Quinn KG Walsh JL , et al. Health care provider barriers to HIV pre-exposure prophylaxis in the United States: a systematic review. AIDS Patient Care STDS 2020; 34(3): 111–123. 32109141 10.1089/apc.2019.0189 PMC7087402

[122] Kaladharan S Daken K Mullens AB , et al. Tools to measure HIV knowledge, attitudes & practices (KAPs) in healthcare providers: a systematic review. AIDS Care 2021; 33(11): 1500–1506. 32964738 10.1080/09540121.2020.1822502

[123] United States Census Bureau. Income and Poverty in the United States: 2020, https://www.census.gov/library/publications/2021/demo/p60-273.html (2022, accessed 13 September 2023).

[124] Moore KL Dell S Oliva MK , et al. Do confidentiality concerns impact pre-exposure prophylaxis willingness in emergency department adolescents and young adults? Am J Emerg Med 2019; 37(6): 1206–1207. 30446420 10.1016/j.ajem.2018.11.015 PMC6509011

[bibr125-20499361241303415] Centers for Disease Control and Prevention (CDC). State laws that enable a minor to provide informed consent to receive HIV and STD Services, https://www.cdc.gov/hiv/policies/law/states/minors.html (2022, accessed 15 August 2023).

[126] Culp L Caucci L. State adolescent consent laws and implications for HIV pre-exposure prophylaxis. Am J Prev Med 2013; 44(1 Suppl. 2): S119–S124. 10.1016/j.amepre.2012.09.044 23253751

[127] Kirby Institute. National update on HIV, viral hepatitis and sexually transmissible infections in Australia: 2009–2018. Kirby Institute, UNSW Sydney,, Sydney, Australia, 2020.

[128] ASHM. PrEP: Australasian society for HIV, viral hepatitis and sexual health medicine, https://www.ashm.org.au/HIV/PrEP/ (2020, accessed 13 September 2023).

[129] Queensland Positive People. Pre-exposure prophylaxis, https://www.qpp.org.au/information/hiv-prevention/prep/#:~:text=PrEP%20is%20available%20on%20the,PrEP%20at%20a%20low%20price (2023).

[130] Office of Infectious Disease and HIV/AIDS Policy HHS. Ready, set, PrEP, https://www.hiv.gov/federal-response/ending-the-hiv-epidemic/prep-program (2022, accessed 13 September 2023).

[131] Hart-Cooper GD Allen I Irwin CE , et al. Adolescent health providers’ willingness to prescribe pre-exposure prophylaxis (PrEP) to youth at risk of HIV infection in the United States. J Adolesc Health 2018; 63(2): 242–244. 29843969 10.1016/j.jadohealth.2018.03.016

[132] Mullins TLK Zimet G Lally M , et al. HIV care providers’ intentions to prescribe and actual prescription of pre-exposure prophylaxis to at-risk adolescents and adults. AIDS Patient Care STDS 2017; 31(12): 504–516. 29211514 10.1089/apc.2017.0147 PMC5724583

[133] Mullins TL Zimet G Lally M , et al. Adolescent human immunodeficiency virus care providers’ attitudes toward the use of oral pre-exposure prophylaxis in youth. AIDS Patient Care STDS 2016; 30(7): 339–348. 27410497 10.1089/apc.2016.0048 PMC4948218

[134] Society for Adolescent Health and Medicine. HIV pre-exposure prophylaxis medication for adolescents and young adults: a position paper of the society for adolescent health and medicine. J Adolesc Health 2018; 63(4): 513–516. 30286903 10.1016/j.jadohealth.2018.07.021

[135] Lopez MI Grant RM Dong BJ. Community pharmacy delivered PrEP to STOP HIV transmission: an opportunity NOT to miss! J Am Pharm Assoc 2020; 60(4): e18–e24. 10.1016/j.japh.2020.01.026 32165026

[136] Schmidt H-MA McIver R Houghton R , et al. Nurse-led pre-exposure prophylaxis: a non-traditional model to provide HIV prevention in a resource-constrained, pragmatic clinical trial. Sex Health 2018; 15(6): 595–597. 30257752 10.1071/SH18076

