# Supplementary material for: Pre-exposure prophylaxis access, uptake and usage by young people: a systematic review of barriers and facilitators
Source: Ther Adv Infect Dis. 2024 Dec 7;11:20499361241303415. doi: 10.1177/20499361241303415 (PMC11624559; doi:10.1177/20499361241303415)
Supplement: sj-docx-1-tai-10.1177_20499361241303415 – Supplemental material for Pre-exposure prophylaxis access, uptake and usage by young people: a systematic review of barriers and facilitators [file sj-docx-1-tai-10.1177_20499361241303415.docx]

**Supplementary Table 1:** search strategy

| **Block 1:** youth/young person terms  “Young people” OR Youth OR “Young adult” OR young OR Adolescen* OR Teen OR Puberty OR Pubescence OR Student OR “High School” OR Sophomore OR Freshman OR College OR University OR Undergraduate |
| --- |
| **AND** |
| **Block 2:** PrEP terms  “Pre-exposure prophylaxis” OR “Preexposure prophylaxis” OR Truvada OR “Biomedical prevention” OR “Biomedical HIV prevention” OR Tenofovir OR Emtricitabine OR “TD/FTC”  MeSH: **“**pre-exposure prophylaxis” |
| **AND** |
| **Block 3:** HIV terms  HIV OR “Human immunodeficiency virus” OR AIDS OR “Acquired Immune Deficiency Syndrome”  MeSH: **“**HIV” |
| **AND** |
| **Block 4:** PrEP use  Use OR usage OR utilisation OR utilization OR uptake OR “patterns of use” OR access OR adherence OR prescrib* OR provide OR Provision OR discontinuation OR cessation OR Barrier* OR facilitat* OR Behavio* OR Knowledge OR Education OR Perception OR Stoppage OR Termination OR Initiat* |
