# Supplementary material for: Pre-exposure prophylaxis access, uptake and usage by young people: a systematic review of barriers and facilitators
Source: Ther Adv Infect Dis. 2024 Dec 7;11:20499361241303415. doi: 10.1177/20499361241303415 (PMC11624559; doi:10.1177/20499361241303415)
Supplement: sj-docx-2-tai-10.1177_20499361241303415 – Supplemental material for Pre-exposure prophylaxis access, uptake and usage by young people: a systematic review of barriers and facilitators [file sj-docx-2-tai-10.1177_20499361241303415.docx]

**Supplementary table 2**: Quality appraisal of included studies using The Mixed Methods Appraisal Tool (MMAT)

|  | **Methodological quality criteria** | | | | | | | | | |
| --- | --- | --- | --- | --- | --- | --- | --- | --- | --- | --- |
| 1. **Qualitative Studies** | | | | | | | | | | |
| **Study Author (year)** | 1.1. Is the qualitative approach appropriate to answer the research question? | | 1.2. Are the qualitative data collection methods adequate to address the research question? | | 1.3. Are the findings adequately derived from the data? | | 1.4. Is the interpretation of results sufficiently substantiated by data? | | 1.5. Is there coherence between qualitative data sources, collection, analysis and interpretation? | |
| Atujuna et al. (2021) | Yes | | Yes | | Yes | | Yes | | Yes | |
| Baron et al. (2020) | Yes | | Yes | | Yes | | Yes | | Yes | |
| Birnholtz et al. (2021) | Yes | | Yes | | Yes | | Yes | | Yes | |
| Camlin et al. (2020) | Yes | | Yes | | Yes | | Yes | | Yes | |
| Crooks et al. (2023) | Yes | | Yes | | No | | Yes | | No | |
| Gailloud et al. (2021) | Yes | | Yes | | Yes | | Yes | | Yes | |
| Hartmann et al. (2021) | Can’t tell | | Yes | | Yes | | Yes | | Yes | |
| Hess et al. (2019) | Yes | | Yes | | Yes | | Yes | | Yes | |
| Marsh & Rothenberger (2019) | Can’t tell | | Can’t tell | | No | | No | | Yes | |
| McKetchnie et al. (2023) | Yes | | Yes | | Yes | | Yes | | Yes | |
| Muhumuza et al. (2021) | Yes | | Yes | | Yes | | Yes | | Yes | |
| Pintye et al. (2021) | Yes | | Yes | | Yes | | Yes | | Yes | |
| Rogers et al. (2021) | Yes | | Yes | | Yes | | Yes | | Yes | |
| Santos et al. (2023) | Yes | | Yes | | Yes | | Yes | | Yes | |
| Shorrock et al. (2022) | Yes | | Yes | | Yes | | Yes | | Yes | |
| Vera et al. (2023) | Yes | | Yes | | Yes | | Yes | | Yes | |
| Zapata et al. (2021) | Yes | | Yes | | Yes | | Yes | | Yes | |
| 1. **Quantitative Studies (Descriptive)** | | | | | | | | | | |
|  | 2.1. Is the sampling strategy relevant to address the research question? | | 2.2. Is the sample representative of the target population? | | 2.3. Are the measurements appropriate? | | 2.4. Is the risk of nonresponse bias low? | | 2.5. Is the statistical analysis appropriate to answer the research question? | |
| Bonett et al. (2021) | Yes | | Yes | | Yes | | Yes | | Yes | |
| Hong et al. (2021) | Yes | | Yes | | Yes | | Can’t tell | | Yes | |
| Macapagal et al. (2020) | Yes | | Yes | | Yes | | Can’t tell | | No | |
| Moskowitz et al. (2021) | Yes | | Yes | | Yes | | Can’t tell | | Yes | |
| Sila et al. (2020) | Yes | | Yes | | Yes | | Yes | | Yrs | |
| Tapsoba et al. (2021) | Yes | | Yes | | Yes | | Yes | | Yes | |
| Tapsoba et al. (2022) | Yes | | Yes | | Yes | | Can’t tell | | Yes | |
| Whitfield et al. (2020) | Yes | | Yes | | Yes | | Can’t tell | | Yes | |
| Zeballos et al. (2022) | Yes | | Yes | | Yes | | Yes | | Yes | |
| 1. **Mixed-Methods Studies** | | | | | | | | | | |
| Barnabee et al. (2022) | Qualitative Assessment | 1.1. Is the qualitative approach appropriate to answer the research question? | | 1.2. Are the qualitative data collection methods adequate to address the research question? | | 1.3. Are the findings adequately derived from the data? | | 1.4. Is the interpretation of results sufficiently substantiated by data? | | 1.5. Is there coherence between qualitative data sources, collection, analysis and interpretation? |
|  |  | Yes | | Yes | | Yes | | Yes | | Yes |
|  | Quantitative Assessment | 2.1. Is the sampling strategy relevant to address the research question? | | 2.2. Is the sample representative of the target population? | | 2.3. Are the measurements appropriate? | | 2.4. Is the risk of nonresponse bias low? | | 2.5. Is the statistical analysis appropriate to answer the research question? |
|  |  | Yes | | Yes | | Yes | | Yes | | Yes |
|  | Mixed Methods Assessment | 3.1. Is there an adequate rationale for using a mixed methods design to address the research question? | | 3.2. Are the different components of the study effectively integrated to answer the research question? | | 3.3. Are the outputs of the integration of qualitative and quantitative components adequately interpreted? | | 3.4. Are divergences and inconsistencies between quantitative and qualitative results adequately addressed? | | 3.5. Do the different components of the study adhere to the quality criteria of each tradition of the methods involved? |
|  |  | Cant’ tell | | No | | No | | No | | Yes |
| Horvath et al. (2019) | Qualitative Assessment | 1.1. Is the qualitative approach appropriate to answer the research question? | | 1.2. Are the qualitative data collection methods adequate to address the research question? | | 1.3. Are the findings adequately derived from the data? | | 1.4. Is the interpretation of results sufficiently substantiated by data? | | 1.5. Is there coherence between qualitative data sources, collection, analysis and interpretation? |
|  |  | Yes | | Yes | | Yes | | Yes | | Yes |
|  | Quantitative Assessment | 2.1. Is the sampling strategy relevant to address the research question? | | 2.2. Is the sample representative of the target population? | | 2.3. Are the measurements appropriate? | | 2.4. Is the risk of nonresponse bias low? | | 2.5. Is the statistical analysis appropriate to answer the research question? |
|  |  | Yes | | Can’t tell | | Yes | | No | | Yes |
|  | Mixed Methods Assessment | 3.1. Is there an adequate rationale for using a mixed methods design to address the research question? | | 3.2. Are the different components of the study effectively integrated to answer the research question? | | 3.3. Are the outputs of the integration of qualitative and quantitative components adequately interpreted? | | 3.4. Are divergences and inconsistencies between quantitative and qualitative results adequately addressed? | | 3.5. Do the different components of the study adhere to the quality criteria of each tradition of the methods involved? |
|  |  | Cant’ tell | | No | | No | | No | | Yes |
| Moskowitz et al. (2020) | Qualitative Assessment | 1.1. Is the qualitative approach appropriate to answer the research question? | | 1.2. Are the qualitative data collection methods adequate to address the research question? | | 1.3. Are the findings adequately derived from the data? | | 1.4. Is the interpretation of results sufficiently substantiated by data? | | 1.5. Is there coherence between qualitative data sources, collection, analysis and interpretation? |
|  |  | Yes | | Yes | | Yes | | Yes | | Yes |
|  | Quantitative Assessment | 2.1. Is the sampling strategy relevant to address the research question? | | 2.2. Is the sample representative of the target population? | | 2.3. Are the measurements appropriate? | | 2.4. Is the risk of nonresponse bias low? | | 2.5. Is the statistical analysis appropriate to answer the research question? |
|  |  | Yes | | Can’t tell | | Yes | | Cant tell | | Yes |
|  | Mixed Methods Assessment | 3.1. Is there an adequate rationale for using a mixed methods design to address the research question? | | 3.2. Are the different components of the study effectively integrated to answer the research question? | | 3.3. Are the outputs of the integration of qualitative and quantitative components adequately interpreted? | | 3.4. Are divergences and inconsistencies between quantitative and qualitative results adequately addressed? | | 3.5. Do the different components of the study adhere to the quality criteria of each tradition of the methods involved? |
|  |  | Yes | | No | | No | | No | | yes |
| Owens et al. (2021) | Qualitative Assessment | 1.1. Is the qualitative approach appropriate to answer the research question? | | 1.2. Are the qualitative data collection methods adequate to address the research question? | | 1.3. Are the findings adequately derived from the data? | | 1.4. Is the interpretation of results sufficiently substantiated by data? | | 1.5. Is there coherence between qualitative data sources, collection, analysis and interpretation? |
|  |  | Yes | | Yes | | Yes | | Yes | | Yes |
|  | Quantitative Assessment | 2.1. Is the sampling strategy relevant to address the research question? | | 2.2. Is the sample representative of the target population? | | 2.3. Are the measurements appropriate? | | 2.4. Is the risk of nonresponse bias low? | | 2.5. Is the statistical analysis appropriate to answer the research question? |
|  |  | Yes | | Yes | | Cant tell | | Yes | | Yes |
|  | Mixed Methods Assessment | 3.1. Is there an adequate rationale for using a mixed methods design to address the research question? | | 3.2. Are the different components of the study effectively integrated to answer the research question? | | 3.3. Are the outputs of the integration of qualitative and quantitative components adequately interpreted? | | 3.4. Are divergences and inconsistencies between quantitative and qualitative results adequately addressed? | | 3.5. Do the different components of the study adhere to the quality criteria of each tradition of the methods involved? |
|  |  | Yes | | No | | No | | No | | Yes |
| Wood et al. (2019) | Qualitative Assessment | 1.1. Is the qualitative approach appropriate to answer the research question? | | 1.2. Are the qualitative data collection methods adequate to address the research question? | | 1.3. Are the findings adequately derived from the data? | | 1.4. Is the interpretation of results sufficiently substantiated by data? | | 1.5. Is there coherence between qualitative data sources, collection, analysis and interpretation? |
|  |  | Yes | | Yes | | Yes | | Yes | | Yes |
|  | Quantitative Assessment | 2.1. Is the sampling strategy relevant to address the research question? | | 2.2. Is the sample representative of the target population? | | 2.3. Are the measurements appropriate? | | 2.4. Is the risk of nonresponse bias low? | | 2.5. Is the statistical analysis appropriate to answer the research question? |
|  |  | Yes | | Yes | | Yes | | Cant tell | | Yes |
|  | Mixed Methods Assessment | 3.1. Is there an adequate rationale for using a mixed methods design to address the research question? | | 3.2. Are the different components of the study effectively integrated to answer the research question? | | 3.3. Are the outputs of the integration of qualitative and quantitative components adequately interpreted? | | 3.4. Are divergences and inconsistencies between quantitative and qualitative results adequately addressed? | | 3.5. Do the different components of the study adhere to the quality criteria of each tradition of the methods involved? |
|  |  | Yes | | No | | No | | No | | Yes |
| Wood et al (2020) | Qualitative Assessment | 1.1. Is the qualitative approach appropriate to answer the research question? | | 1.2. Are the qualitative data collection methods adequate to address the research question? | | 1.3. Are the findings adequately derived from the data? | | 1.4. Is the interpretation of results sufficiently substantiated by data? | | 1.5. Is there coherence between qualitative data sources, collection, analysis and interpretation? |
|  |  | Cant tell | | Yes | | Yes | | Yes | | Yes |
|  | Quantitative Assessment | 2.1. Is the sampling strategy relevant to address the research question? | | 2.2. Is the sample representative of the target population? | | 2.3. Are the measurements appropriate? | | 2.4. Is the risk of nonresponse bias low? | | 2.5. Is the statistical analysis appropriate to answer the research question? |
|  |  | Yes | | Yes | | Yes | | Cant tell | | Yes |
|  | Mixed Methods Assessment | 3.1. Is there an adequate rationale for using a mixed methods design to address the research question? | | 3.2. Are the different components of the study effectively integrated to answer the research question? | | 3.3. Are the outputs of the integration of qualitative and quantitative components adequately interpreted? | | 3.4. Are divergences and inconsistencies between quantitative and qualitative results adequately addressed? | | 3.5. Do the different components of the study adhere to the quality criteria of each tradition of the methods involved? |
|  |  | Yes | | No | | No | | No | | Yes |
